# A Geometrical Model for DNA Organization in Bacteria

**DOI:** 10.1371/journal.pone.0013806

**Published:** 2010-11-03

**Authors:** Mathias Buenemann, Peter Lenz

**Affiliations:** 1 Department of Physics and Center for Theoretical Biological Physics, University of California San Diego, La Jolla, California, United States of America; 2 Fachbereich Physik and Zentrum für Synthetische Mikrobiologie, Philipps-Universität Marburg, Marburg, Germany; Loyola University Medical Center, United States of America

## Abstract

Recent experimental studies have revealed that bacteria, such as *C. crescentus*, show a remarkable spatial ordering of their chromosome. A strong linear correlation has been found between the position of genes on the chromosomal map and their spatial position in the cellular volume. We show that this correlation can be explained by a purely geometrical model. Namely, self-avoidance of DNA, specific positioning of one or few DNA loci (such as origin or terminus) together with the action of DNA compaction proteins (that organize the chromosome into topological domains) are sufficient to get a linear arrangement of the chromosome along the cell axis. We develop a Monte-Carlo method that allows us to test our model numerically and to analyze the dependence of the spatial ordering on various physiologically relevant parameters. We show that the proposed geometrical ordering mechanism is robust and universal (i.e. does not depend on specific bacterial details). The geometrical mechanism should work in all bacteria that have compacted chromosomes with spatially fixed regions. We use our model to make specific and experimentally testable predictions about the spatial arrangement of the chromosome in mutants of *C. crescentus* and the growth-stage dependent ordering in *E. coli*.

## Introduction

Eukaryotic cells have an elaborate machinery that organizes the genome over several length scales. As far as is known, bacteria do not posses the proteins required for such a sophisticated organization. On small length scales DNA is a stiff polymer. This makes DNA organization in small systems (such as viruses) particularly difficult. The length scale on which the DNA appears stiff is the persistence length defined as the decay length of tangent-tangent correlations [for a short introduction into the basic concepts and notions of polymer physics see Supplemental Information (SI) text S1]. On length scales much larger than the persistence length DNA, however, adapts a random coil conformation, i.e. a conformation where the monomers are oriented randomly giving rise to a global shape without specific secondary structures. But nevertheless the bacterial genome can have a highly regular spatial structure as has recently been found in *C. crescentus* [1]. The spatial structure of the chromosome leads to a strong linear correlation between the position of a gene on the chromosome and its position in the subcellular volume. In *C. crescentus* swarmer cells (that are in the non-replicating G1 state) this arrangement has the characteristic feature that origin (*ori*) and terminus (*ter*) are positioned at opposite cell poles. To realize such an arrangement in newborn swarmer cells *ori* and *ter* have to undergo a complex coordinated movement during replication: after duplication the new *ori* moves to the opposite (new) pole while *ter* relocates to midcell. This gives rise to a mirror image conformation of the chromosome once *ter* is duplicated [2].

It has been demonstrated that *ori* positioning is realized by anchoring the chromosome to the pole by the PopZ protein [3], [4]. However, the mechanisms that give rise to positioning of *ter* and to the linear arrangement of the chromosome in *C. crescentus* cells have so far not been revealed. Also the physiological role of the spatial arrangement of the chromosome is unknown. It has been speculated that transcriptional processes such as transertion (that leads to localization of genes that encode for trans-membrane proteins or proteins that are transported through the membrane) could influence the chromosomal arrangement [5].

There is evidence that other bacteria also spatially organize their chromosomes (for a review see Ref. [6]). The most prominent example is *E. coli* that exhibits complex chromosomal organization and dynamics [7]. The current understanding of these phenomena is incomplete and a consistent picture is only slowly emerging. Part of the problem is that there are evident contradictions in some of the reported observations (that might partly be caused by differences in the way cell cultures are synchronized and differences in growth conditions). In particular, very different chromosome arrangements have been reported in the literature. Some studies report that in newborn cells *ori* and *ter* are localized at opposite poles [8], [9]. Upon initiation of replication *ori* and *ter* change their positions and move towards midcell and stay side by side aligning parallel to the cell axis (i.e. the axis connecting the poles) [9]. Other studies report that upon initiation of replication only *ori* has a well-defined position in midcell and *ter* is drawn to midcell only during replication [10], [11]. After duplication, the two *E. coli ori* move in opposite directions towards the cell poles and the bulk of the chromosome assumes a left-*ori*-right-left-*ori*-right configuration [10], [12], [13], [14] (where left and right refer to the left and right chromosome arm, respectively). In this case the newborn cells have *ori* placed at midcell with a broadly distributed position of *ter*.

The mechanisms that lead to this complex growth-phase dependent chromosome movement are unknown. Possibly, many different and overlapping processes are involved. An important contribution could come from active migration of chromosomal domains (where the actin-like MreB protein [15] or the migS sequence [16] could play a role). Furthermore, the DNA replication process itself might direct the different chromosome arms into the designated cell half. In this context it has been speculated that replication might occur at spatially fixed positions (“replication factories”) [17]. Evidence against this has been reported in Ref. [13] but further studies are required to fully resolve this question.

Here we demonstrate that confinement of chromosomal domains to specific cellular positions has a strong influence on the spatial arrangement of the chromosome in the cell. In particular, the positioning of *ori* and *ter* to opposite cell poles in *C. crescentus* gives rise to the striking linear correlation found in Ref. [1]. For *E. coli* we make predictions about the growth-stage dependence of the spatial arrangement of the chromosome.

## Results

We theoretically analyze the basis of chromosomal organization in bacteria. To do so we employed stochastic Monte Carlo computer simulations to generate ensembles of bacterial DNA configurations obeying the following constraints: (A) the DNA has fixed (prescribed) length, (B) the DNA lies inside a prescribed cellular volume, and (C) *ori* and *ter* have fixed positions.

### Non-compacted DNA

**Figure 1:**
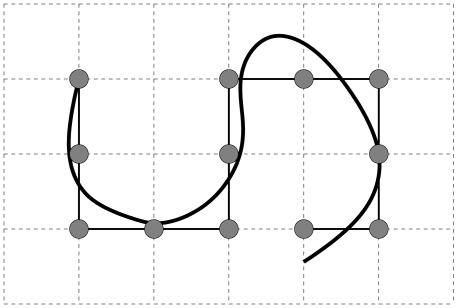
Representation of DNA as Random Walk. In the simulations the DNA configuration (black solid line) is represented by a random walk on a three-dimensional cubic lattice. The random walk can be thought of as a discrete set of connected beads. The lattice constant is given by the Kuhn length 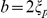 (which is twice the persistence length 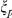). With this step size the directions of two sequential steps are completely uncorrelated. In the simplest picture the random walk may intersect itself, since the diameter of DNA is much smaller than the grid size.

We started with the simplest model of the bacterial chromosome where DNA is described as a non-compacted semi-flexible polymer (for details see SI text S1) confined to the cell. Thus, effects arising from proteins that compact DNA (such as HU, H-NS and SMC proteins [18]) are not taken into account. In the simulations the cellular volume is discretized and represented by a three-dimensional cubic lattice. The chromosome is represented as a random walk on this lattice. The step size is given by the Kuhn length 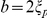, which is twice the persistence length 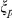 of DNA. In this way it is guaranteed that the random walk and the semi-flexible polymer it represents have the same statistics. DNA has a diameter of ∼2nm and can cross a 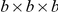 sized box several times without intersecting with itself, see Fig. 1. Thus, in the lattice representation of non-compacted DNA self-avoidance of the random walk does not have to be taken into account, i.e. the random walk may cross a lattice site more than once.

In the following, we first present simulation results for *C. crescentus*. In the simulations the implemented cells have height 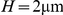 and cross section 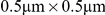. For simplicity we focus on *C. crescentus* swarmer cells where the single chromosome is in the nonreplicative G1 state [19]. The DNA length is then 1.3mm (4.02Mbp). In those instances where 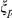 is not varied we set 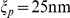 to mimic the *in vivo* effects of supercoiling and the reduction in persistence length by DNA binding proteins (that are not compaction proteins), for details see discussion. For this value the *C. crescentus* cells were represented as boxes of dimensions 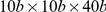 (DNA length = 26000*b*). In the following, we first assume that *ori* and *ter* have fixed positions at the center of the lower and upper cell wall (at 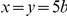). As mentioned, *ori* is anchored by PopZ, but the molecular mechanism that leads to *ter* positioning is still unknown. According to the experimental findings of Ref. [1] (see also the discussion below) the apparent distance of *ori* and *ter* from the cell walls is quite large and we have correspondingly set the *z*-positions for *ori* and *ter* respectively to 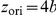 and 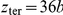 (where the cell axis is chosen to be the *z*-axis). For random walks without self-avoidance it is sufficient to analyze the configuration of only half the chromosome. In the following we assume that *ori* is at 6 o'clock and *ter* at 12 o'clock on the chromosomal map. Then, it is sufficient to analyze the segment connecting *ori* and *ter* (i.e. the clockwise connection from 6 o'clock to 12 o'clock). In this way phase space can be fully explored since this segment has the same statistics as the segment from *ter* to *ori* (i.e. the clockwise connection from 12 o'clock to 6 o'clock). Random walks of length 13000*b* were generated in a two-step process. First, we constructed a single random walk that obeyed the above constraints (A)–(C). Starting from this initial configuration the phase space was sampled using an algorithm proposed by Madras, Orlitsky, and Shepp (MOS) [20], for details see Materials and Methods. In this way we generated 10^6^ random walks of length equal to half of the genome length of *C. crescentus*.

**Figure 2:**
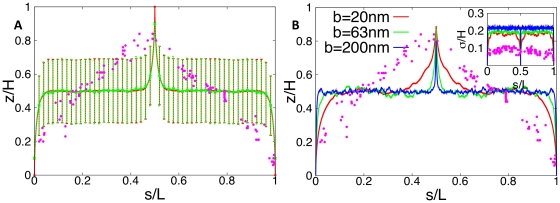
Average subcellular position of genes as function of their position on the chromosome in *C. crescentus* as obtained from numerical simulations of non-compacted DNA. The subcellular position is obtained by averaging the *z*-positions of 10^6^ different (non self-avoiding) random walks that represent an ensemble of non-compacted chromosomes of *C. crescentus* confined to the cell volume represented by a lattice of size 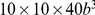 (corresponding to a volume of 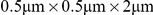 for 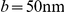). The position on the chromosome is parameterized by the contour length *s* (measured in units of DNA length *L*). (A) Walk of length 26000*b* representing the genome. *Ori* and *ter* lie on the cell axis at opposite poles. Their distance from the bottom and top cell walls is 0*b* (red curve) and 4*b* (green curve). Error bars represent the standard deviations between the different DNA configurations sampled in the 10^6^ runs. Dots represent the experimental data from Ref. [1]. (B) Same plot as in (A) but for different Kuhn lengths (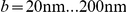). DNA length and cell volume are kept constant. Here, the standard deviations between the individual realizations are rescaled and shown as function of the position on the chromosome.

To compare the chromosome configurations (that are represented by the random walks) with the experimental data from Ref. [1] we analyzed the correlation between the position on the chromosome and the position along the cell axis. To do so, we calculated the average *z*-position as function of contour length of the chromosome for these 10^6^ random walks, see green curve in Fig. 2A. Evidently, the strong linear correlation found in the experimental data (dots in Fig. 2A) is not reproduced by the simulation, where the average DNA configuration remains for most of the steps in the middle of the cell (at 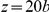). More precisely, the middle of the cell is already reached after ∼2000 steps. However, the DNA is not equally distributed in the cell, as can be seen by calculating the standard deviation from the mean position (at 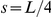). The above distribution has a standard deviation of 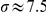 implying that 68% of the chromosome can be found in the region between 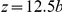 and 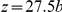. For comparison an equidistribution between 0 and 40 (where the DNA is uniformly distributed in the cellular volume) has a standard deviation 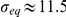. Thus, the entropic repulsion of the DNA from the walls of the confining geometry leads to a stronger localization of the chromosome in midcell.

To analyze the influence of the proximity to the wall on the statistics we have also implemented a second ensemble with different *z*-positions for *ori* and *ter* with 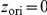 and 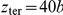, respectively. The red curve in Fig. 2A shows the corresponding statistics of DNA conformations. For this case the statistics of the random walk only slight improves. Although it takes a few more steps until the average *z*-position settles at its mean value, the DNA is still mainly localized in the middle of the cell.

We have also checked that these findings are a general feature of the representation of the chromosome by a non self-avoiding walk and are independent of the precise value of the persistence length, see Fig. 2B. Indeed, a reduction in 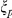 only leads to a slight increase in the number of steps it takes to reach the plateau. But still 90% of all steps have a mean *z*-position in the middle of the cell. Larger values of the persistence length make the statistics even worse since the average random walk reaches the middle earlier and stays there longer.

**Figure 3:**
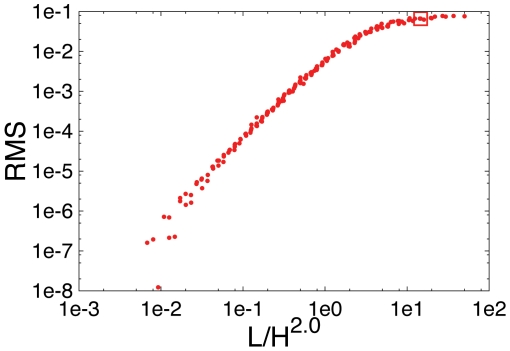
Root mean square deviation of the average (non-compacted) DNA configuration from a strictly linear configuration for different DNA lengths and different sizes of the confining volume. The figure shows the root mean square deviation (RMS) from the strictly linear configuration that connects *ori* and *ter* by a line (see Eqs. [1] and [2]) as a function of DNA length *L* and distance *H* between *ori* and *ter* for cell shapes with the aspect ratio of *C. crescentus*. RMS is a function of 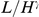 with 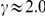. The square indicates the data point for *L* = 26000, *H* = 40 corresponding to the parameter values of *C. crescentus*.

As mentioned supercoiling of DNA leads to a reduction of persistence length. It also reduces the radius of gyration (∼30% according to Ref. [21]) of the DNA and thus compacts DNA and effectively reduces its length *L*. We therefore checked whether such a smaller *L* would have an effect on our results. However, we found that the average DNA configuration still looked like the ones shown in Fig. 2. We next analyzed how strong the reduction in *L* would need to be in order to obtain the experimentally observed linear arrangement of the average DNA configuration. To do this in a systematic way we quantified the deviations of the simulation results from the DNA configuration that linearly connects *ori* and *ter* by calculating the root mean square deviation
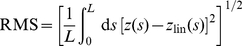
(1)of the average DNA shape from the (idealized) linear configuration parameterized by
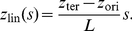
(2)We calculated the RMS for a wide range of DNA lengths *L*. Furthermore, to take into account that the persistence length 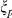 could also be further reduced by supercoiling we also varied the size of the confining volumes (which is measured in units of the Kuhn length 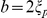). To do so, we implemented cell shapes with the aspect ratio of *C. crescentus* (given by elongated boxes of size 
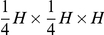
) and varied *L* = 400…40000 and *H* = 40…400. In all cases, *ori* and *ter* were located at the centers of the cell walls thus having also distance *H*. Interestingly, we find that all RMS calculated from Eq. [1] for the different combinations of *L* and *H* collapse onto a single curve if plotted as function of 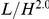, see Fig. 3. Furthermore, this function increases with increasing 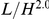 and one sees that only for very short DNA sequences the RMS is close to zero indicating a straighter configuration. For example, for *C. crescentus* the linear relationship would not even be obtained if the DNA were 100 times shorter. From the 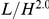 values at which these configurations occur it becomes clear that the supercoiling-induced reduction of the radius of gyration cannot be responsible for this.

### Compacted DNA

Fig. 3 shows that linear DNA configurations can only be expected for small DNA length. Such a reduction in length could be explained by the actions of proteins that compact DNA and which are not taken into account in the above model. To investigate this further we developed a model for compacted DNA in which DNA is, again, treated as a semi-flexible polymer. However, here the compaction proteins locally give the DNA the shape of a sphere with diameter 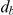. In polymer physics such a spherical arrangement is called a “blob”.

In the following we assume that a blob typically contains one DNA-loop (induced by H-NS, HU, FIS and perhaps TktA [22]) that could be further compacted by supercoiling [21]. In *E. coli* the average loop size is ∼10kB [23]. The blob radius of such an average loop is given by the radius of gyration 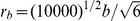, where 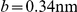 is the length of one base. Thus, 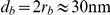 and for *E. coli* the whole genome could be represented by ∼400 of these blobs. However, a significant fraction of loops is smaller than ∼10kB [23]. Furthermore, some loops might be less compact and not all regions of the chromosome necessarily belong to a loop. In the following we therefore assume that there are ∼2000 blobs with average diameter 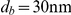. We assume that *C. crescentus'* chromosome is organized in a similar way. As we show below our findings do not depend on these specific assumptions.

To take these effects into account we represented the compacted DNA by a chain of blobs each having the diameter 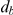. In order to simulate this model one has again to use a discretization scheme where DNA configurations are represented by random walks on a lattice. However, here each step of the random walk represents a rather extended piece of DNA (and not only a DNA segment of the length of the lattice spacing). Therefore, the lattice spacing has to be chosen accordingly (i.e. the lattice size has to be set to 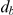) and the self-avoidance of the random walk has to be taken into account. This makes the numerical investigation much more difficult since an exact enumeration of self-avoiding walks (SAWs) is only feasible for chain lengths up to ∼20–30 blobs which is insufficient for this investigation. To generate an ensemble of self-avoiding walk we therefore had to use an approximation scheme based on the MOS method [20], for details see Materials and Methods.

Using this method we calculated the average configuration of self-avoiding walks (representing compacted DNA) for different blob sizes. Since in our description of compacted DNA size and number of blobs are unknown parameters we systematically varied their values and checked for which parameter range our model gives good agreement with the experimental findings. In doing so, we considered two different ensembles: ensemble (i) in which the DNA density per blob is constant and ensemble (ii) in which the number of blobs is constant. In ensemble (i) we varied the blob diameter 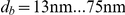. DNA content per blob was set to 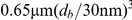 and the length of the simulation box thus changes from 154 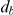 to 27 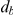, while the DNA length changes from 24578 blobs to 128 blobs. In ensemble (ii) the number of blobs per DNA (2000) is assumed to be constant. The blob radius is varied in the range 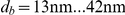. Each blob contains DNA of length 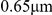, independent of its radius. The physiological differences between these two ensembles are explained in the discussion section.

**Figure 4:**
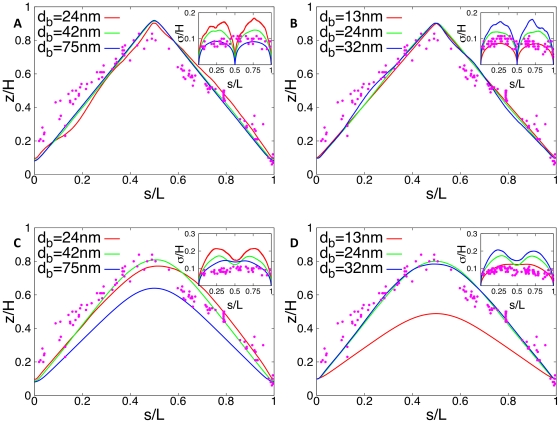
Average subcellular position of genes as function of their position on the chromosome in *C. crescentus* as obtained from numerical simulations of compacted DNA. The *z*-position of an average chromosome configuration was calculated from our model in which compacted DNA is represented by a chain of blobs. The position on the chromosome is parameterized by the contour length *s* (measured in units of DNA length *L*). The configurations shown are for different blob diameters. In Fig. A a constant density of DNA per blob has been assumed, in Fig. B a constant number (2000) of blobs. The insets show the (rescaled) standard deviations from the mean configurations as function of *s*. Dots are experimental data from Ref. [1]. The fixed positions of *ori* and *ter* (given by 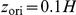 and 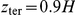 where *H* is the length of the cell) have been adjusted to minimize the differences between experimental data and the predictions of the model. Figs. C and D are for the same blob parameters as A and B, respectively. However, in C and D only *ori* has a fixed position at 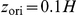, while *ter* is free to move. One should note that because of the additional freedom in moving *ter* the DNA configuration shows a different dependence on the blob size than with fixed *ter*.

Furthermore, we varied the (fixed) positions of *ori* and *ter* (inside the cellular volume represented by a box of size 
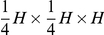
) to minimize the differences between the predictions of the model and the experimental data from Ref. [1]. Best agreement was found for 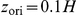 and 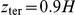. The corresponding (average) DNA configurations together with the standard deviations obtained for these parameter values are shown in Fig. 4. As can be seen the predictions of the theoretical model agree pretty well with the experimental data. The linear correlation between spatial position and position on the chromosome is clearly reproduced for a range of blob diameters 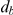 = 24nm to 75nm in ensemble (i) and 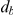 = 13nm to 32nm in ensemble (ii). In particular, linear correlations are found in both ensembles. However, as one can see from Fig. 4 in both ensembles a symmetric DNA configuration is predicted (in which the segment from *ter* to *ori* is a mirror image of the segment from *ori* to *ter*) while the experimentally found DNA configuration is asymmetric (genes with a fixed chromosomal distance from the origin have a somewhat larger spatial distance from the *ori*-pole if they are on the segment from *ori* to *ter*). Possible explanations are discussed below.

To make sure that in ensemble (ii) the results do not depend on our choice of number of blobs (2000) we have also systematically varied this quantity (at fixed blob diameter 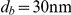). As shown in Fig. S1 the chromosome configuration is nearly linear for blob numbers ranging from 200 to 2000. To keep the DNA length fixed at *L* = 1.3mm the DNA length per blob has to be adjusted (from 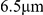/blob to 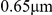/blob). However, the DNA configurations do not depend on this density and the results shown in Fig. S1 can also be interpreted as average chromosome configurations with lengths ranging from 0.13mm to 1.3mm (and constant DNA length of 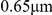/blob). This shows that the proposed mechanism for DNA arrangement is robust and works in a large range of parameter values.

As can be seen from Fig. 4, in the average DNA configuration genes that are located left of the origin (i.e. that are on the segment from *ori* to *ter*) show a positive correlation between position on the chromosome and position in the cell: the further away from *ori* a gene is positioned on the chromosomal map the further away from the *ori*-pole at 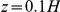 the gene is found in the cellular volume. Similarly, the genes located right of the origin (i.e. that are on the segment from *ter* to *ori*) show a negative linear correlation. This is a direct consequence of the fact that two opposite positions on the chromosome (*ori* and *ter*) are kept at fixed opposite positions in the cell. We were wondering whether it is really necessary to position both *ori* and *ter* at fixed cell positions to get these linear and symmetric chromosomal configurations. To do so, we analyzed *C. crescentus* cells that had only *ori* fixed to the flagellated pole by anchoring by PopZ [3], [4]. To our surprise, we found even in this case linear configurations, see Figs. 4C and 4D. The main difference is that without positioning of *ter* the chromosomal arrangement seems to be less robust with respect to variations in the blob radius.

**Figure 5:**
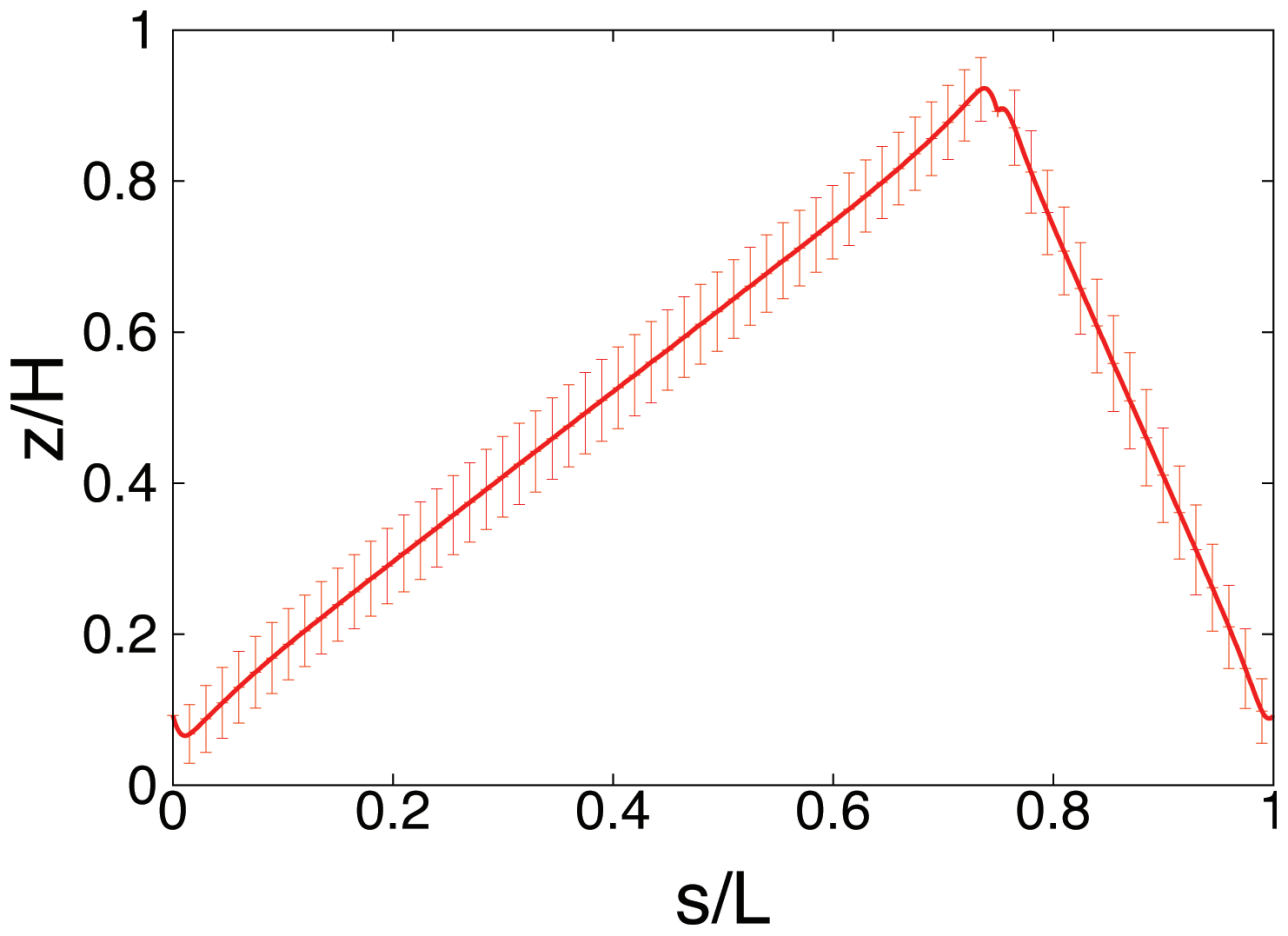
Average DNA configuration in a *C. crescentus* mutant cell where *ori* is located at 6 o'clock and *ter* at 3 o'clock on the chromosome. In the simulations the chromosome is represented by a self-avoiding chain of blobs with diameter 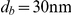. The chain consists of 2000 blobs: 1500 blobs for the segment connecting *ori* and *ter* and 500 blobs for the segment connecting *ter* and *ori*. Cell size is 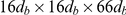. The error bars denote standard deviations from mean position.

A comparison of Figs. 4A and 4B with Figs. 4C and 4D reveals that the position of *ter* is almost identical in the two situations. This implies, that even though *ter* appears *in vivo* at a specific position one cannot conclude that this position is fixed by, e.g., anchoring to the pole. In fact, the positioning of *ori* together with the steric repulsion between the topological domains could lead to the positioning of *ter*. There is a way to experimentally probe this in a (fictive) *C. crescentus* mutant whose terminus has an altered position on the chromosome (that these regions indeed can be moved on the chromosome has been shown recently in Ref. [24]). In the mutant, *ori* would be located at 6 o'clock and *ter* at 3 o'clock (as opposed to 12 o'clock in the standard configuration). Again, *ori* is positioned at 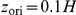. Our model predicts that the chromosomal configuration now strongly depends on the spatial positioning of *ter*. If there is no direct mechanism that fixes the position of *ter* (i.e. if the *ter* position only depends on the DNA configuration) then this mutant will show the same average chromosome configuration as shown above in Fig. 4C and 4D. However, if *ter* position is fixed at 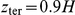 then in the mutant the correlation between the position of the genes on the chromosome and within the cell is altered: it is still linear but now 75% of all genes (those on the segment from *ori* to *ter*) have a positive correlation between contour length *s* (i.e. distance from *ori* on the chromosome) and *z*-position in the cellular volume. Only 25% of all genes have a negative correlation between *s* and *z*, see Fig. 5.

### Compacted DNA: *E. coli*

The above results show that, according to our model, the ordering of DNA in *C. crescentus* is solely of geometrical origin: The strong linear correlation between the position of the genes on the chromosome and their spatial position is a direct consequence of the facts that DNA is compacted and that either *ori* or *ori* and *ter* have fixed positions at opposite poles. But these are rather general features that are also fulfilled in other bacteria, such as in *E. coli*. As mentioned, here the localization patterns are somewhat more complicated, but *ori* and *ter* still have (growth phase-dependent) fixed positions. We used our model to make predictions about the DNA configurations that arise from such a growth-phase dependent positioning of *ori* and *ter* in *E. coli*.

**Figure 6:**
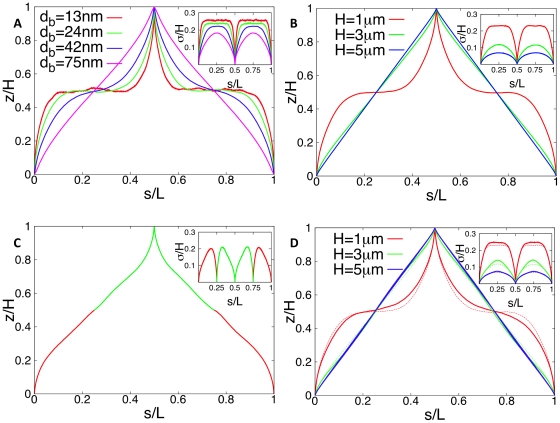
Average subcellular position of genes as function of their position on the chromosome in newborn *E. coli* cells as obtained from numerical simulations of compacted DNA. The figure shows the *z*-position of an average chromosome configuration as function of the contour length *s*. In our model a chain of blobs represents the compacted DNA. The position on the chromosome is parameterized by *s* (measured in units of DNA length *L*). In figures A, B, and D *ori* and *ter* are positioned at the cell poles (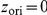 and 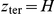). The configurations shown in figure A are for a cellular volume of 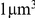 and different blob diameters by assuming a constant density of DNA of 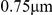 per blob. The dependence on the volume at constant DNA density is shown in figure B. Cell shapes are varied at constant cross section but different length 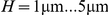
(corresponding to H = 33…165 blobs). The largest newborn cell has a volume of 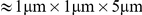. Figure C shows a DNA configuration in a cell with two chromosomes (shown in different colors) just prior to cell division. The two *ori* have fixed positions at the cell poles, the two *ter* are kept at midcell. The contour length is measured along the path left (chromosome #1)-right (chromosome #2)-left (chromosome #2)-right (chromosome #1). Data shown are for a volume of 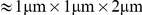 and each chromosome is represented by 2000 blobs. In this way the cellular DNA density remains constant and that the length of compacted DNA per blob (given by 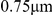 DNA per blob) is independent of the volume. A DNA configuration in these faster growing cells at an earlier stage of the cell cycle is shown in figure D. Here, the cell contains an additional DNA strand whose ends are anchored in the midplane of the cell mimicking the geometry of the chromosome after half the replication time [when the replication forks are located at 3 o'clock and 9 o'clock on the mother chromosome (solid lines)]. The presence of additional DNA makes the linear correlation stronger. For comparison the DNA configurations without daughter DNA are shown (dashed lines). Parameter values are as in figure B. The insets show the (rescaled) standard deviations from the mean configurations as function of *s*.

To test these predictions we implemented in the simulations a confining cell volume of 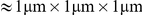
(corresponding to the cell volume of a newborn *E. coli* cell with doubling time>60min [25]) containing a chromosome of 1.5mm length [corresponding to a genome size of 4.6Mbp (*E. coli* K12)]. We first focused on the results of Ref. [9] where in newborn cells *ori* and *ter* are localized at opposite poles. Since no precise data are available we assumed that both *ori* and *ter* have fixed positions at 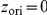 and 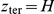
(where *H* denotes the length of the bacterium). However, from our results for *C. crescentus* we expect that this choice has only a (minor) influence on the DNA configuration but not on the linear correlation (between spatial position and position on the chromosome) itself. Furthermore, as standard configuration we used (as estimated above) 2000 blobs of radius 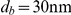 (with a density of 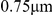 DNA per blob). The results obtained in ensemble (i) are shown in Fig. 6A. As one can see, in *E. coli* there is only a linear correlation (between position on the chromosome and position in the cellular volume) for sufficiently large blob diameters (i.e. not too strong compaction). For small blobs there is a clear correlation between the position on the chromosome and in the cell but significant deviations from the linear relation occur. Similar results are obtained in ensemble (ii), see Fig. S2.

Width and length of *E. coli* are strain-dependent [26]. In particular, the cells used in Ref. [9] are more elongated than the ones used in the simulations above. To see if the cellular geometry has an influence on our findings we systematically varied the cell volume at constant DNA length by varying the length *H* of the cells. In doing so, we implemented, as observed experimentally [27], cell shapes of constant cross section but different length 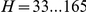 (blobs), where *H* = 165 represents the largest newborn cell with a volume of 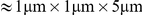. As can be seen from Fig. 6B for a large range of volumes there is a strong correlation between the position of genes on the chromosome and inside the cellular volume. In particular, the chromosomal configuration becomes more linear in more elongated cells.

The size of *E. coli* also depends on growth rate. Faster growing cells are bigger and for doubling times faster than 60 minutes one has to take into account that they have more DNA. Generally, this gives rise to a complex chromosomal topology with multiple replication forks and, depending on growth rate, multiple origins and termini (whose localization patterns have been observed in Ref. [28]). As explained in detail in the discussion our current algorithm is not suitable to analyze this complex scenario. However, to check if geometrical ordering can also work in cells that have more than one chromosome, we analyzed the situation just prior to cell division where the cell has two fully replicated chromosomes. As shown in Ref. [9] in this case the two *ter* are in midcell while the two *ori* are at opposite cell poles. These two chromosomes can be represented by a single random walk (that is twice as long as the random walk representing a single chromosome) whose midpoints (where the two *ter* meet) and the ends (the two *ori*) have fixed positions. With DNA length we increased the volume of the cell such that the DNA density remains constant [29]. As can be seen from Fig. 6C the correlation now extends over both chromosomes. It is also interesting to note that the positioning of *ori* and *ter* also leads to a significant demixing of the two chromosomes (see standard deviations of the average configuration shown in the inset of Fig. 6C).

In deriving these results we have assumed that the length of compacted DNA per blob (given by 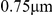 DNA per blob) is independent of the volume. This assumption would, e.g., be fulfilled if the number of compaction proteins increases during the cell cycle (and thus increases with volume) or with growth-rate. However, similar results are found if the number of compaction proteins is constant. In this case the blob radius increases with increasing volume due to the lower concentration of compaction proteins, see Fig. S3. Furthermore, we have checked that also for *E. coli* our results do not depend on the number of blobs, see Fig. S4 that shows that a quasi-linear DNA configuration is found for blob numbers ranging from 68 to 632.

As mentioned, with the current algorithm we cannot make theoretical predictions about the chromosomal arrangements if several replication forks are moving along the DNA. However, we expect that in this case the presence of additional DNA makes the linear correlation stronger. An indication that this is true can be seen from Fig. 6D, that shows the configuration of a chromosome in presence of a DNA strand whose ends are anchored in the midplane of the cell mimicking the geometry of the chromosome after half the replication time (when the replication forks are located at 3 o'clock and 9 o'clock on the mother chromosome).

**Figure 7:**
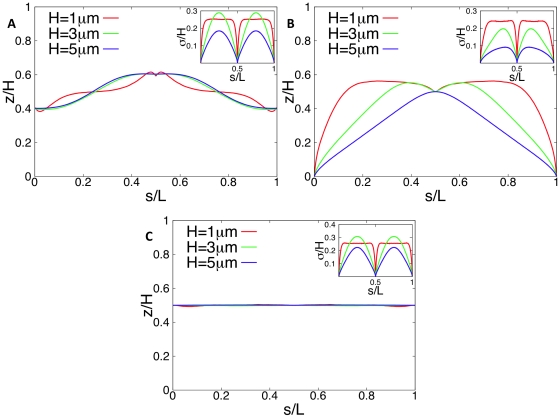
Average subcellular position of genes for different *ori* and *ter* positions in *E. coli* as obtained from numerical simulations of compacted DNA. The figures show the *z*-position of an average chromosome configuration as function of the contour length *s*. Cell length varies from 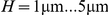, i.e. 33 to 165 blob diameters (with a blob diameter of 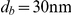) at constant DNA density, i.e. the number of blobs is kept constant at 2000. In Fig. A *ori* and *ter* are positioned in midcell (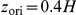 and 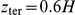), in Fig. B *ori* is at the cell pole and *ter* is positioned in midcell (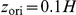 and 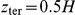), and in Fig. C both *ori* and *ter* are in midcell (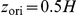 and 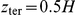 with 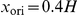 and 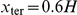). The insets show the (rescaled) standard deviations from the mean configurations as function of *s*.

The above results on the arrangement of a single chromosome can only be expected for newborn *E. coli* cells under the conditions of Ref. [9], where *ori* and *ter* are localized at opposite poles [30]. As mentioned, upon initiation of replication *ori* and *ter* move towards midcell. This influences the DNA arrangement in the cell. If *ori* and *ter* arrange in the way reported in Ref. [9] (i.e. are fixed at 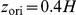 and 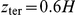) then in the average configuration the remaining chromosome indeed localizes between *ori* and *ter*, see Fig. 7A. However, as the large standard deviations indicate in the individual realizations significant deviations from the population average occur.

The findings of Ref. [31] suggest that compaction of DNA together with *ori* and *ter* positioning might also be important for chromosome segregation after DNA replication. To test this we have calculated the average DNA configuration of a chromosome whose *ori* is kept close to the cell pole while *ter* is kept at a fixed position in the middle of the cell. As one can see from Fig. 7B, the average DNA configuration is indeed mainly localized in the cell half that contains *ori*. This arrangement is found for all relevant volumes of *E. coli*.

As explained in the introduction, other studies found a different positioning pattern of *ori* and *ter*. In Refs. [10], [11] *ori* had a well-defined position in midcell and *ter* is drawn to midcell only during replication. We were wondering whether this positioning would give rise to the observed left-*ori*-right orientation of the chromosome [12]. To test this we positioned *ori* and *ter* in midcell at same height (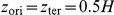) but with different x-positions (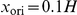 and 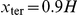). Thus, in this case the line connecting *ori* and *ter* is perpendicular to the cell axis. As shown in Fig. 7C the corresponding average DNA configuration stays in the middle of the cell, implying that the left-*ori*-right and the right-*ori*-left configurations are equally likely. This suggests that additional mechanisms are required to conserve the orientation of the chromosome.

Finally, in a mutant that has *ter* at 3 o'clock similar DNA configurations should be experimentally observable as for *C. crescentus* provided that *ori* and *ter* are fixed to specific positions close to the cell poles. Fig. S5 shows an example for a newborn *E. coli* cell under the conditions of Ref. [9].

## Discussion

Our results show that the correlation between the position of a gene on the chromosome and inside the cellular volume can be explained by a purely geometrical model. It is a consequence of the positioning of *ori* and *ter* to specific spatial positions and the elastic properties of DNA.

As mentioned, on small length scales DNA is a stiff polymer, while on large length scales it adapts a random coil conformation. These properties make a detailed microscopic theoretical description of DNA difficult. Therefore, to study its conformations one has to consider coarse-grained models where the structures on length scales below 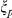 are neglected. One possibility is to model the DNA as a “freely jointed chain” of independent segments of length 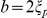 (where *b* is the Kuhn length). Then, the direction of each segment is completely uncorrelated to its preceding segments. The chain is also allowed to intersect itself. In absence of external fields and walls the energy of the chain is zero and all configurations are equally likely.

Here, we are interested in the statistical properties of DNA under the constraints that either *ori* or *ori* and *ter* have fixed positions in the cell. Generally, the incorporation of these geometrical constraints into a theoretical model for chromosome configurations is non-trivial. It is simply impossible to analytically solve the equations that describe the elastic properties of DNA under these constraints. Therefore, one is limited to numerical investigations that require using a discretized description. One possibility is to analyze the DNA configurations on a lattice. To do so, the shape of the DNA is approximated by a random walk on a three-dimensional cubic lattice with grid spacing 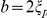, see Fig. 1. Although the angle distribution between neighboring segments is not continuous (but rather restricted to the directions of the lattice vectors of the grid) such models have shown to have similar properties as continuous chains. For example, experimentally measured force distance relations of stretched polymer molecules are well reproduced by such models [32].

In the cell DNA interacts with many proteins. Some of them modulate the structure of DNA and can lead to significant compaction of the nucleoid. In our model these local structures (typically extending over a few hundred basepairs) are represented as ‘blobs’, i.e. spheres containing DNA under the influence of a single or several compaction proteins. As mentioned above, the picture we have in mind is that proteins such as H-NS, HU, FIS etc. bind to certain regions of the chromosome and give it a compact local structure such that the chromosome consists of a chain of compact units. For our model we do not need to make any specific assumptions on how these local structures are organized and packaged. The detailed molecular mechanisms are not important here since the goal of our analysis is to see if the combination of boundary conditions, namely the positioning of *ori* and *ter*, and this local structuring is sufficient to explain the experimentally observed organization on the global scale. For example, additional effects such as supercoiling of DNA [21] and polycation-dependent DNA-DNA interactions might also contribute to compaction [33]. Additionally, proteins such as structural maintenance of chromosomes complexes (SMC) can further modulate the chromosome structure on larger scales [34]. Even if the blobs emerge randomly by, say, stochastic binding of compaction proteins, the theoretical predictions are robust with respect to the associated variations in blob number and average radius (see discussion below).

First, however, we have analyzed if compaction is necessary at all to get the correlation between spatial localization and chromosomal position. As explained above, in the absence of compaction proteins, DNA can simply be represented as a non self-avoiding walk on a lattice with lattice constant 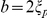 since on large length scales semiflexible polymers behave effectively as freely jointed chain with this length [35].

For the analysis of our results the persistence length is a crucial parameter. *In vitro* it has a value 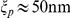 [36], [37]. *In vivo*, supercoiling (together with the binding of non-compaction proteins) leads to a reduced stiffness 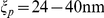 [38] justifying our choice of 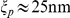 in the analysis of non-compacted DNA. For compacted DNA where compaction proteins such as HU also influence 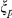, Skoko et al. [39] find *in vitro* that 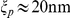 for a HU concentration close to the intracellular value. Typically one has 1 HU per 300 bp, together with a binding site length of 10–40bp [40] this corresponds to an occupancy around 10%. For such a density of proteins on DNA a ∼50% reduction of 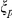 is also expected theoretically [41].

In the absence of compaction proteins the average DNA configuration (represented by an average non self-avoiding random walk with step size 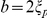 on a lattice) is very different from that observed for *C. crescentus* in Ref. [1]. As can be seen from Fig. 2, the DNA is mainly localized in the middle of the cell. We have shown that these findings are general features of the model and do not depend on the specific (fixed) position of *ori* and *ter*, the value of the persistence length, DNA length or the size of the confining volume (see Fig. 3).

Thus, the main finding of this analysis is that the linear relationship between positions of the genes on the chromosome and their spatial location within the cell cannot be explained for non-compacted chromosomes (represented by non self-avoiding walks on a lattice). Also, supercoiling does not significantly increase this correlation neither via reduction of persistence length nor compaction by reduction of radius of gyration (which is of the order of ∼30% [21]).

**Figure 8:**
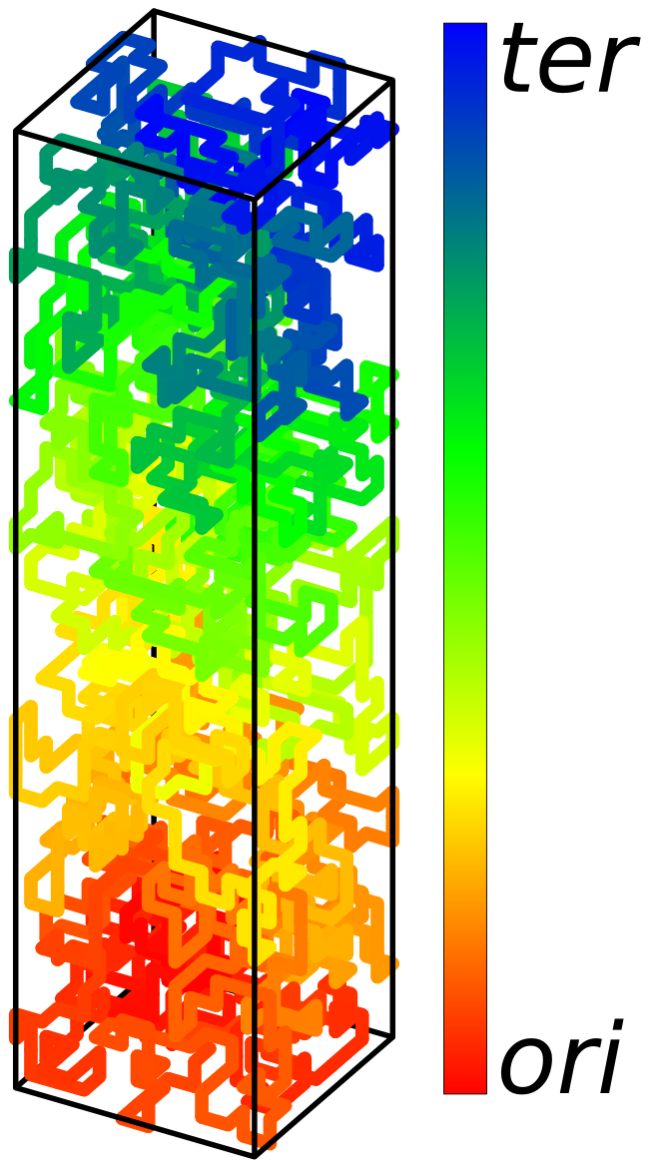
Typical DNA configuration of an individual *C. crescentus* cell belonging to a population that has an average DNA configuration showing the linear correlation between position of genes on the chromosome and position in the cell. The DNA configuration was calculated from our model for compacted DNA. The DNA was represented as a self-avoiding walk on a lattice with 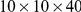 sites (representing a cellular volume of 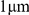). The lattice spacing is equal to the blob diameter 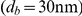. The chromosome then consists of 2000 blobs. The color coding represents the distance from *ori* and *ter*: gene positions close to *ori* are shown in red, gene positions close to *ter* are shown in blue. Intermediate regions on the *ori* to *ter* and on the *ter* to *ori* segment are shown in green.

The other striking feature of the spatial arrangement of the non-compacted chromosome is that there are strong variations between the individual realizations (as indicated by the large standard deviations from the mean curve, see Fig. 2). If spatial organization of the chromosome has a physiological role, one expects a small variance and thus a positioning that only varies slightly between different realizations. At this point it is important to mention that in order to show that an ensemble of DNA configurations has certain properties (such as the strong correlation between position of the genes on the chromosome and within the cell) a sufficiently large sample of possible configurations has to be analyzed. Ideally, the sample includes an appropriate representation of all possible configurations such that the sample average represents the population average. However, DNA organization is a stochastic process exhibiting cell-to-cell variations. Therefore, the statistical analysis then only can show that the average configuration has the desired properties and that the variation between the individual realizations are small. The mean configuration corresponds to the population average and correspondingly this average is measured if the DNA configurations would be analyzed in many cells simultaneously as done in Ref. [1]. But nevertheless the individual cells belonging to a population exhibiting a linear correlation between the position on the chromosome and the position in the cell volume typically have a DNA configuration that deviates from the average configuration. Fig. 8 shows a typical example for a chromosome configuration in an individual *C. crescentus* cell. As can be seen the shown structure is rather ring-like than intertwined.

In our model for compacted DNA the chromosome configuration is represented by a self-avoiding walk. Each step is given by a spherical blob of diameter 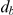 as a local representation of the compacted structure. Since every step of the random walk now represents a rather extended part of the DNA self-avoidance of the walk has to be taken into account. This makes the calculation of the statistical properties of an ensemble of self-avoiding walks highly challenging even for the rather small number of steps needed here to represent the compacted chromosome. An exact enumeration of walks is impossible and an approximation scheme has to be used by which an unbiased sample of configurations can be generated. As explained in Materials and Methods we have used the MOS algorithm [20] to generate such an ensemble of random walks.

As mentioned, from measurements in *E. coli* we expect that typical blob diameters are of the order 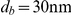 and that there are ∼2000 blobs per genome. We also assumed that in *C. crescentus* these parameters take similar values. However, our findings are independent of these assumptions indicating that the proposed mechanism is robust and works for a large range of parameter values provided that both *ori* and *ter* have fixed positions. More specifically:

- As shown in Figs. 4A, 4B and 6A the DNA configurations predicted by our model show a strong correlation between spatial and genome localization provided that the blobs are large enough. Generally, for a given blob size the correlation is somewhat stronger in *C. crescentus* than in slowly growing newborn *E. coli* cells with a volume of 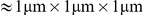 (and the *ori* and *ter* localization pattern of Ref. [9]), due to the more elongated shape of *C. crescentus*. For *C. crescentus* the relationship is nearly perfectly linear for 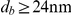, for *E. coli* for 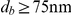 (where all blobs have a constant density of DNA) suggesting that the chromosome is less compacted in slowly growing *E. coli* cells. This robustness with respect to 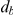 is important since there is a certain arbitrariness in our definition of the blob radius (caused by the lack of detailed information on how DNA is compacted on the molecular scale). For example, a blob could also contain several DNA loops and *in vivo* there is a distribution of blob sizes [23]. In this sense the above standard blob with diameter 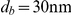 can be seen as the minimal unit of compacted DNA.
- The linear correlation also holds for a large range of blob numbers: for *C. crescentus* for 200 to 2000 blobs, for *E. coli* for 200 to 600 blobs, see Figs. S1 and S4. Robustness with respect to variations in the number of blobs (and to variations in the blob radius) is important since (in our model) these are not regulated quantities but rather the outcome of stochastic events (such as the unspecific binding of the compaction proteins to DNA). In this way, ensemble (ii) (where the number of blobs is constant) describes a scenario where the number of DNA compaction proteins is constant but their binding sites show a cell-to-cell variation. Similarly, in ensemble (i) (where the DNA density per blob is constant) the number of compaction proteins varies but their binding sites are fixed. There is not enough experimental data available to decide whether ensemble (i) or (ii) better describes the *in vivo* dependencies.
- Linear DNA configurations are also found in a large range of cell volumes, see Fig. 6B. This is important for *E. coli* that shows a ∼10-fold change in volume with growth rate.
- With increasing DNA content the linear arrangement of the chromosome becomes stronger, as can be seen from Figs. 6C and 6D. Furthermore, the geometrical ordering also works for a large range of chromosome lengths (ranging from *L* = 1.5mm to 3mm) indicating that our proposed mechanism is applicable to different bacteria.

By comparing the results of our first model (non-compacted chromosome represented by a non self-avoiding random walk) with those of the second model (compacted chromosome represented by a self-avoiding walk) it becomes clear that compaction of the chromosome is an essential ingredient to obtain the observed linear correlation. In fact, self-avoidance only contributes little to this ordering. More specifically, the linear correlation could also be obtained from our first model provided that the chain is short enough. This can be seen from Fig. 3 where it is shown that the linear correlation becomes stronger as the length of the chromosome decreases. As we have shown above, the experimental data cannot be explained by a model that includes only self-avoidance (that for example could be induced by electrostatic repulsion between the DNA) but not a mechanism that effectively reduces the length of the random walk representing the chromosome. In our second model this reduction of length is assumed to be the result of the compaction of the chromosome.

The importance of chromosomal compaction for spatial positioning in *E. coli* was also demonstrated in Ref. [42]. There, a ‘fluctuating filament model’ was introduced in which the chromosome is approximated as a confined elastic filament with constant DNA packing density. In contrast to our model fluctuations of the chain are taken into account and it was shown that the variance in locus positioning is well explained by the model provided that the packing density is sufficiently high. At this point it is worth mentioning that compaction should also affect the mobility of the chromosome. In Ref. [43] it was shown that fluorescently labeled chromosomal loci diffuse significantly slower than expected from the Rouse-like behavior of a polymer under confinement. A contribution to this subdiffusive motion of the chromosome arises from a pin-and-pivot mechanism (where, at a given instance, only some of the segments are free to move) [44] which is the pattern of motion one expects for a confined chain of blobs.

Although our model is able explain the general linear correlation between positioning of genes on the chromosome and their location in the cellular volume there are small differences between the experimental data and the theoretical predictions in Fig. 4. We checked that these deviations are not caused by the curvature of *C. crescentus* that is not taken into account in our simulations (data not shown). Thus, the differences are either due to experimental errors or they indicate that there are additional mechanisms that influence the ordering. For the latter case, we speculate in Fig. S6 what kind of chromosomal arrangement would give better agreement with the experimental data. As shown, it appears that the segment from *ori* to *ter* stays closer to *ter* than to *ori* and that the segment from *ter* to *ori* stays closer to *ori* than to *ter*. During replication such an arrangement might be favorable for the separation of the two segments.

Experimentally, it is quite evident that in *C. crescentus ter* has a specific position in the cell. However, the mechanism of this localization is unknown. For example, it could be caused by the action of a protein that anchors *ter* to the cell membrane. However, in our analysis we also find a linear chromosome arrangement if only *ori* has a fixed position. In this case *ter* is free to move but its spatial position is confined by the configuration of the remaining parts of the chromosome, see Figs. 4C and 4D. This indicates that the experimentally observed fixed position of *ter* does not imply that *ter* is indeed anchored. However, if the position of *ter* is not fixed the chromosomal configuration is more sensitive to the size-distribution of topological domains. At this point it should be mentioned that in order to get linear chromosomal arrangements that are in agreement with the experimental data of Ref. [1] it is necessary to keep the position of at least one DNA locus fixed.

In *E. coli* the chromosome arrangement strongly depends on the growth-stage. Newborn cells show (at least under the conditions of Ref. [9] and sufficiently large topological domain sizes) a linear configuration similar to that observed in *C. crescentus*. Under the same experimental conditions *ori* and *ter* then move toward midcell upon initiation of replication. The results of our simulations suggest that in this case the positioning of *ori* and *ter* leads (on average) to a more confined arrangement of the chromosome (in the space between *ori* and *ter*), see Fig. 7A. In this way the space extending from the poles to the positions of *ori* respectively *ter* contains less DNA thus possibly making space for the newly replicated chromosomal arms. However, because of the large standard deviations of this configuration the individual realizations might deviate significantly from the average one leading to a less confined chromosome.

Once replication is finished, the old *ori* moves back to the pole, while *ter* stays in midcell leading to a chromosome that is mainly confined to one cell half, see Fig. 7B. In this way *ori* and *ter* positioning might contribute to chromosome segregation. Finally, our results indicate that the observed conservation of the left-*ori*-right orientation of the chromosome is not a consequence from positioning of *ori* and *ter* suggesting that additional mechanisms are responsible for this.

Our model can, in principle, be generalized to describe chromosomal configurations in faster growing cells that have several replication forks. However, from a computational point of view this is highly challenging since the set of transformations by which the random walks are generated (for details see Materials and Methods) have to guarantee that mother and daughter strands stay connected at the replication fork by preserving the self-avoidance of the chain. This is a non-trivial task if the site of the replication fork itself is transformed. Experimentally it has been seen that under these conditions complicated localization patterns or *ori* and *ter* emerge [28]. It is unknown if tethering of the additional *ori* and *ter* is required to explain the observations. However, from our above finding that positioning of *ori* is already sufficient to fix the position of *ter* we do not expect that is necessarily the case.

## Materials and Methods

In our model for compacted DNA the chromosome is represented by a self-avoiding random walk. Each step represents a DNA-blob. To construct an ensemble of such walks we start from a self-avoiding rectangular loop of minimal length passing through *ori* and *ter*. This walk is then elongated by breaking a randomly chosen bond and replacing it by a hook, see Fig. S7. In this way two steps are added to the walk. If one of the chosen sites was already occupied before, the transformation is rejected to ensure that the walk remains self-avoiding. The procedure is repeated until the chain has the desired length. The volume constraint is implemented by allowing only bead positions inside the volume. Similarly, chromosome configurations with fixed *ori* but free *ter* are realized by requiring that the random walks start and end at *ori*.

To get ensembles of self-avoiding walks that have good statistics one has to appropriately select the set of transformations that operates on the random walk, for details see SI text S1. We have used the scheme introduced by Madras, Orlitsky, and Shepp [20] that include “bead flips” [45] and “crankshafts” [46] moves, see Figs. S8 and S9. The MOS algorithm produces an ergodic ensemble of self-avoiding walks of fixed length and with fixed ends constraints. We compared the results of the MOS scheme with results obtained from exact enumeration to check that the approximation scheme produces good statistics, for details see SI text S1 and Figs. S10, S11 and S12.

## Supporting Information

Text S1 (0.20 MB DOC)

Figure S1
**Dependence of the average DNA configuration in *C. crescentus* on the number of blobs as obtained from numerical simulations of compacted DNA.** The *z*-position of an average chromosome configuration was calculated from our model in which compacted DNA is represented by a chain of blobs. The position on the chromosome is parameterized by the contour length *s* (measured in units of DNA length *L*). The configurations shown are for different number of blobs with diameter 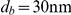. *ori* and *ter* have fixed positions at 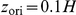 and 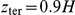.(0.73 MB TIF)

Figure S2
**Average subcellular position of genes as function of their position on the chromosome in newborn *E. coli* cells as obtained from numerical simulations of compacted DNA.** The figure shows the *z*-position of an average chromosome configuration as function of the contour length *s*. In our model a chain of blobs represents the compacted DNA. Configurations shown are for different blob diameters by assuming a constant number (2000) of blobs. *ori* and *ter* are positioned at opposite cell poles (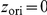 and 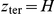). The insets show the (rescaled) standard deviations from the mean configurations as function of *s*.(0.74 MB TIF)

Figure S3
**Dependence of the average chromosome configuration in newborn *E. coli* cells on the cellular volume.** The *z*-position (as function of the contour length *s*) of an average chromosome configuration was calculated from our model in which compacted DNA is represented by a chain of blobs. In the figure the volume is varied by changing the length of the cells (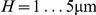) by keeping the aspect ratio of the cross section fixed. Chromosome length is varied together with the volume such that the DNA density in the volume remains constant. Furthermore, the number of compaction proteins is assumed to be growth-rate independent. The chromosome is represented by 2000 blobs with a volume-dependent diameter (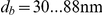). *Ori* and *ter* are positioned at opposite cell poles (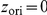 and 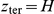). The insets show the (rescaled) standard deviations from the mean configurations as function of *s*.(0.22 MB TIF)

Figure S4
**Dependence of the average DNA configuration on the number of blobs for newborn *E. coli* cells as obtained from numerical simulations of compacted DNA.** The figure shows the *z*-position of an average chromosome configuration as function of the contour length *s*. The configurations shown are for different number of blobs with diameter 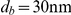. *Ori* and *ter* are positioned at opposite cell poles (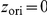 and 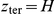).(0.76 MB TIF)

Figure S5
**Average DNA configuration in a newborn *E. coli* mutant cell where *ori* is located at 6 o'clock and *ter* at 3 o'clock on the chromosome.** Both *ori* and *ter* are located at opposite cell poles (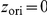 and 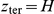). In the simulations the chromosome is represented by a self-avoiding chain of blobs with diameter 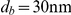. The chain consists of 2000 blobs: 1500 blobs for the strand connecting *ori* and *ter* and 500 blobs for the strand connecting *ter* and *ori*. Cell size is 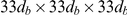 (corresponding to 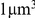). The error bars denote standard deviations from mean position.(0.69 MB TIF)

Figure S6 **Possible DNA configuration in *C. crescentus*.** Schematic illustration of a DNA configuration that could give rise to the observed small deviations from the linear correlation between position on the chromosome and in the cellular volume. The *ori* to *ter* strand preferably stays close to *ter*, while the *ter* to *ori* strand stays close to *ori*. The figure on the right shows the corresponding *z*-position of the genes as function of their position on the chromosome (solid curves). The dashed curve represents a perfect linear correlation. In the experimental data the deviation from the linear correlation is much smaller than shown here.
(0.14 MB TIF)

Figure S7 **Construction scheme for SAWs.** Starting from a minimal self-avoiding walk that connects *ori* and *ter* a randomly chosen bond is deleted. If the (randomly chosen) neighboring lattice sites are free (gray) they are incorporated into the random walk. In this way, the chain is closed again and *ori* and *ter* remain at their original positions.
(0.65 MB TIF)

Figure S8
**Flip- and crankshaft-transformations for SAWs.** A flip of the gray bead converts the random walk 2122 (a) into 2212 (b). In (c) the gray bead is not allowed to flip. This problem is resolved by a crankshaft move that transforms 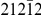 into 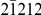 (d). Here, the random walk is represented by a string of symbols where, e.g. 1 represents “up”, 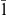 “down”, 2 “right”, and 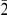 “left”.(0.70 MB TIF)

Figure S9
**3-dimensional crankshaft-transformation.** Three-dimensional crankshaft-transformations introduce new symbols into the random chain. In the example shown, the random walk 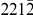 is transformed into 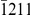, thus replacing a 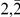 pair by a 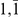 pair. This is accomplished by the following sequence of transformations (shown from left to right): a crankshaft move (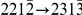), followed by two bead flips (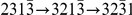) and another crankshaft move (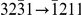). All moves operate on the gray beads. The black beads are fixed.(0.60 MB TIF)

Figure S10
**Direct comparison of the BF and the MOS method.** Starting from a common initial configuration 1000 BF- and MOS-moves were performed to obtain 50000 different SAWs of length 2000 confined to a volume of 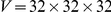 steps. The figure shows the mean *z(s)* curves obtained by these methods by averaging over the 50000 samples. The mean standard deviations are 0.07 (BF) and 0.19 (MOS) showing that the BF method produces walks that stay much closer to the initial configuration.(1.04 MB TIF)

Figure S11
**Comparison of the MOS algorithm with exact enumeration by using the radial density of self-avoiding walks.** Figure A shows the density of self-avoiding loops generated by the MOS algorithm. The length of the random walks was 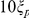 for the strand connecting origin and terminus and 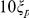 for the strand connecting terminus and origin. The distance between terminus and origin is 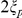 (along the *z*-axis). Figure B shows the difference between the densities of the random ensemble and the exact ensemble. The MOS method reproduces the exact curves quite well except for a small region between *ori* and *ter* that is slightly underrepresented.(0.24 MB TIF)

Figure S12 **Comparison of the MOS algorithm with exact enumeration by using the statistics of the *z*-positions.** The figure shows average *z*-positions of self-avoiding random walks as calculated with the MOS algorithm (red curve) and by systematic enumeration (green curve). Data are for the same parameter values as Fig. S11.
(0.68 MB TIF)
