# Supplementary material for: A Geometrical Model for DNA Organization in Bacteria
Source: PLoS One. 2010 Nov 3;5(11):e13806. doi: 10.1371/journal.pone.0013806 (PMC2972204; doi:10.1371/journal.pone.0013806)
Supplement: Text S1 — (0.20 MB DOC) [file pone.0013806.s001.doc]

**Supporting Information**

Buenemann and Lenz

**SI Theoretical Background**

**Theoretical models for elastic properties of DNA**

There are several theoretical models that allow the description of the elastic properties of DNA and other polymers. Typically these models successfully predict observable quantities either at the single polymer or ensemble level. Important examples include the theoretical predictions of stress-strain relations of polymers in external fields or scattering amplitudes from neutron scattering experiments on polymer coils [1].

For the purpose of our study, the worm like chain model (WLC) is the most appropriate description for the properties of DNA. Here, the elastic energy *E* of DNA (or any other semiflexible polymer) just depends on its curvature

$E=k_{b}T\frac{\xi_{p}}{2}\int_{0}^{L} \left|\partial_{s}\vec{t}(s)\right|^{2} \text{d}s ,$ [S1]

where *T* is the temperature, *L* the length and $\overrightarrow{t}$is the tangent vector to the curve that represents the DNA configuration. In Eq. [S1] and in the sequel the configuration is parameterized by the contour length *s*. The shape of the polymer is obtained by integration over the local tangent vectors $\overrightarrow{t}(s)$. The radius of gyration that measures the end-to end distance is then given by

[S2]

An important property of worm like chain polymers is that the correlation between two tangent vectors decays exponentially with their distance *l*,

[S3]

Here, denotes an ensemble average over many different realizations. The characteristic decay length for the tangential correlations is given by the persistence length $\xi_{p}$of the WLC. The WLC model however is only useful to describe the behavior on small length scales. For polymers longer than several persistence lengths, it is often not necessary to take into account the details of the conformations on the scale of the persistence length [1]. In this case, one typically considers coarse-grained models where the structures on length scales below $\xi_{p}$ are neglected. Then, the DNA is modeled as a “freely jointed chain” of independent segments each having length $b=2\xi_{p}$, where *b* is the Kuhn length. With this choice of segment length the freely jointed chain has the same mean-square end-to-end distance and the same contour length as the polymer that it represents. The direction of each segment is completely uncorrelated to its preceding segment. In particular, in such a description the chain may intersect itself. In absence of an external field, the energy of the chain is zero in each configuration and all configurations are equally likely.

Generally, such a description is useful in explaining properties of bulk polymers. For example, the force distance behavior of stretched polymer molecules has been successfully explained by such a model [2]. The analysis of confined polymers is much more complex since the incorporation of the corresponding geometrical constraints is by no means easy. Generally this can only be done numerically by representing the polymer configuration by a random walk. In the simplest description this is a random walk on a cubic lattice. Then, in contrast to freely jointed chains, the angle distribution between neighboring segments of the random walk is no longer continuous but restricted to the directions of the lattice vectors of the grid.

Random walk models find many applications in physics: they are typically used to model Markov processes, i.e. processes that do not show a history-dependence. The most prominent examples are diffusion processes, in particular Brownian motion. Here, the motion of a small particle is over-damped (i.e. all inertial contributions can be neglected) and solely determined by thermal fluctuations, which do not depend on the past trajectory of the particle. Similarly, segments in a DNA configuration that are further apart than the persistence length $\xi_{p}$are uncorrelated. In particular, over this distance the tangent directions of the DNA configuration become randomized. Thus, the shape of the DNA can be divided into segments of length $b=2\xi_{p}$and approximated by a random walk on a cubic lattice with grid spacing *b*, see Fig. 1 of the main text. DNA has a diameter of $\sim2\text{nm}$and can therefore cross a $b\times b\times b$ sized box several times without intersecting itself. Hence, the random walk representing the DNA on the coarse grained lattice may visit a lattice site more than once.

**Random walks**

Computationally, ensembles of non-self avoiding walks (non-SAW) are easy to generate, but the additional constraints (that, as mentioned, are required to describe the effects of confining geometries or the fixed positions of the ends) impose a challenging problem. In order to obtain an ensemble in which all random walks have equal weights one has in principle to (i) generate unconstrained walks of a given length and (ii) discard all walks that do not meet the given constraints. However, this method becomes already infeasible at relatively small lengths since the probability to arrive at a certain point after a certain number of steps decreases dramatically with the total number of random steps. Because of the central limit theorem, the probability to arrive at a point $\vec{x}$ in *N* steps is given by

[S4]

Thus, with increasing walk length the probability to encounter a given point $\vec{x}$ goes asymptotically to zero as $P(\vec{x})\sim N^{-3/2}$. The probability to find a random walk is even further reduced if the average radius of gyration is smaller than the confining volume. Clearly, generating an ensemble of self-avoiding walks (SAWs) in this way is practically impossible, since in three dimensions the number of (unconstrained) SAWs of length *N* scales with $N^{1/6}$ [1].

In our simulations we generated ensembles of SAWs and non-SAWs in a two-step process. First, we constructed an initial walk that met the constraints. Subsequently, we applied a set of geometrical transformations that (i) respect the constraints and (ii) sample the configuration space ergodically. In the following section this procedure is described in detail with special focus on SAWs. The method can be adapted to non-SAW in a straightforward way.

**Self-avoiding walks**

Computationally, it is quite difficult to construct spatially confined SAWs of given length with fixed ends. One possibility is to use the following construction scheme: (i) the starting configuration is a self-avoiding rectangular loop of minimal length passing through 2 given points (in our case representing *ori* and *ter*). (ii) The chain is elongated by breaking a randomly chosen bond and replacing it by a hook, see Fig. S7. In this way two beads are added to the chain. If any of the newly chosen sites was occupied before, the transformation is rejected to ensure that the walk remains self-avoiding. The procedure is repeated until the chain has the desired length. To obey the volume constraint only bead positions inside the confining volume are allowed.

In an ensemble of SAWs constructed in this way the RMS (see Eq. [1] of the main text) is close to 0 for all *L* and *H* (data not shown), arising artificially from the unbiased choice of bonds at which new hooks are introduced. Furthermore, regions between *ori* and *ter* are underrepresented compared with an ensemble based on exact enumeration (data not shown). Berg and Foerster [3] proposed that one can generate an ensemble of unconstrained SAWs with a Boltzmann probabilistic weight by applying flip- and crankshaft-transformations to an initial SAW, see Fig. S8. In the following, we refer to this method as BF method.

The statistics of the bead flip method can be understood qualitatively. To do so, it is convenient to represent the random walk by a string of symbols where, e.g., 1 represents “up”, $\bar{1}$ “down”, 2 “right”, and $\bar{2}$ “left”. For SAWs moves represented by $i\bar{i}$are forbidden. In a bead flip two neighboring symbols change position, see Fig. S8. In this way, bead flips generate a diffusion of symbols along the chain. However, in a situation like the one represented by the string$212\bar{1}2$ flipping is not allowed for SAWs, see Fig. S8C. Such a configuration represents an impenetrable barrier for diffusion. To resolve this one has to introduce crankshaft moves. This transforms the random walk $212\bar{1}2$ into $2\bar{1}212$allowing thus the symbols $\bar{1}$and $1$to diffuse, see Fig. S8D. For unconstrained SAWs (with free ends) the first and last beads are also allowed to flip. In this way new symbols are introduced into the random chain. It has been shown that the introduction of new symbols is essential to generate a proper ensemble of SAWs (see Ref. [3] and references therein).

Here, however, we consider constrained random walks and new symbols cannot be introduced at the fixed ends. To be able to do so, the crankshaft moves have to be generalized to three dimensions. Fig. S9 shows an example where a sequence of crankshaft moves and bead flips transforms the random walk $221\bar{2}$ into $\bar{1}211$, thus altering the total amount of symbols $1,\text{ }\bar{1}$ and $2,\text{ }\bar{2}$. In this way new symbols can be introduced into the chain even if the ends are kept fixed.

**MOS algorithm**

The BF method includes only a small subset of possible moves that transform a SAW into different SAW. A more general procedure for the construction of SAWs with fixed ends and fixed length has been introduced by Madras, Orlitsky, and Shepp [4]. They showed that the set of allowed bead flips and crankshaft moves of the BF method has to be extended to yield an ergodic ensemble.

The MOS algorithm consists of three transformations operating on segments of a SAW, which leave the constraints unaltered: (i) reflection, (ii) inversion, and (iii) interchange of bonds. Note, that a bead flip is just a reflection transformation of a 2-step segment. Similarly, in 2D a crankshaft move is an inversion transformation of a 3-step segment. In 3D a crankshaft motion is equivalent to an interchange transformation of a 3-step segment. Therefore, bead flips and crankshaft moves are included in the MOS algorithm.

For very long segments MOS transformations become increasingly improbable due to self-avoidance and the MOS algorithm operates mainly on small segments (like the BF method). However, the MOS algorithm allows the tying and untying of knots, which cannot be done by bead flips and crankshaft transformations alone. In fact, sampling of the ensemble with the BF method yields mean *z(s)*-curve with a mean standard deviation close to zero) indicating that every walk segment is stuck close to its initial position. Fig. S10 shows a direct comparison of the results obtained by the BF and the MOS method.

As mentioned, the MOS method extends the BD method to yield an ergodic ensemble. However, ergodicity only implies that all allowed paths can be constructed by the stochastic method, but does not necessarily yield exact ensemble statistics. To check the validity of the MOS algorithm we recursively constructed all 562,845,352 self-avoiding loops of length $2\times10\xi_{p}$(i.e. self-avoiding walks with 20 steps). The loops start at the origin and have to reach the terminus after exactly 10 steps (of total length$10\xi_{p}$). After another 10 steps they must return to the origin. Terminus and origin were positioned at $z=6\xi_{p}$and $z=4\xi_{p}$, respectively. The volume was chosen to be so large that it did not confine the random walk. A stochastic ensemble of $10^{6}$walks was generated by the MOS method starting from symmetric, self-avoiding loops of length $2\times4\xi_{p}$.

In Fig. S11 we compare the density of walks as calculated with the MOS method and the density obtained from the systematic enumeration. Fig. S11A shows the radial density of walks for the stochastic method. Here, the density of a lattice site is defined as the number of walks passing through that point normalized by the total number of walks in the ensemble. The difference of the densities for the random ensemble and the exact ensemble is shown in Fig. S11B. As the figures show the MOS method is able to reproduce the exact curves quite well except for a small region between *ori* and *ter* that is slightly underrepresented. This can also be seen from Fig. S12 where the average *z*-position is plotted as a function of the DNA contour length. After the first step a stochastic path is encountered more frequently below the origin (or above the terminus) compared with the average in the exact ensemble.

**REFERENCES**

1. de Gennes PG (1979) Scaling Concepts in Polymer Physics.

2. Marko J, Siggia ED (1995) Stretching DNA. Macromolecules 28: 8759 - 8770.

3. Hilhorst HJ, Deutch JM (1975) Analysis of Monte Carlo results on the kinetics of lattice polymer chains with excluded volume. J Chem Phys 63: 5153-5161.

4. Madras N, Orlitsky A, Shepp LA (1990) Monte Carlo generation of self-avoiding walks with fixed endpoints and fixed length. J Stat Phys 58: 159- 183.

**FIGURE LEGENDS**

**SI Supporting Figures**

**Fig. S1.** **Dependence of the average DNA configuration in *C. crescentus* on the number of blobs as obtained from numerical simulations of compacted DNA.**

The *z*-position of an average chromosome configuration was calculated from our model in which compacted DNA is represented by a chain of blobs. The position on the chromosome is parameterized by the contour length *s* (measured in units of DNA length *L*). The configurations shown are for different number of blobs with diameter $d_{b}=30\text{nm}$. *ori* and *ter* have fixed positions at $z_{\text{ori}}=0.1H$and $z_{\text{ter}}=0.9H$.

**Fig. S2.** **Average subcellular position of genes as function of their position on the chromosome in newborn *E. coli* cells as obtained from numerical simulations of compacted DNA.**

The figure shows the *z*-position of an average chromosome configuration as function of the contour length *s.* In our model a chain of blobs represents the compacted DNA. Configurations shown are for different blob diameters by assuming a constant number (2000) of blobs. *ori* and *ter* are positioned at opposite cell poles ($z_{\text{ori}}=0$and $z_{\text{ter}}=H$). The insets show the (rescaled) standard deviations from the mean configurations as function of *s*.

**Fig. S3.** **Dependence of the average chromosome configuration in newborn *E. coli* cells on the cellular volume.**

The *z*-position (as function of the contour length *s*) of an average chromosome configuration was calculated from our model in which compacted DNA is represented by a chain of blobs. In the figure the volume is varied by changing the length of the cells ($H=1...5\mu\text{m}$) by keeping the aspect ratio of the cross section fixed. Chromosome length is varied together with the volume such that the DNA density in the volume remains constant. Furthermore, the number of compaction proteins is assumed to be growth-rate independent. The chromosome is represented by 2000 blobs with a volume-dependent diameter ($d_{b}=30...88\text{nm}$). *Ori* and *ter* are positioned at opposite cell poles ($z_{\text{ori}}=0$and $z_{\text{ter}}=H$). The insets show the (rescaled) standard deviations from the mean configurations as function of *s*.

**Fig. S4.** **Dependence of the average DNA configuration on the number of blobs for newborn *E. coli* cells as obtained from numerical simulations of compacted DNA.**

The figure shows the *z*-position of an average chromosome configuration as function of the contour length *s*. The configurations shown are for different number of blobs with diameter $d_{b}=30\text{nm}$. *Ori* and *ter* are positioned at opposite cell poles ($z_{\text{ori}}=0$and $z_{\text{ter}}=H$).

**Fig. S5. Average DNA configuration in a newborn *E. coli* mutant cell where *ori* is located at 6 o'clock and *ter* at 3 o'clock on the chromosome.** Both *ori* and *ter* are located at opposite cell poles ($z_{\text{ori}}=0$and $z_{\text{ter}}=H$). In the simulations the chromosome is represented by a self-avoiding chain of blobs with diameter $d_{b}=30\text{nm}$. The chain consists of 2000 blobs: 1500 blobs for the strand connecting *ori* and *ter* and 500 blobs for the strand connecting *ter* and *ori*. Cell size is $33d_{b}\times33d_{b}\times33d_{b}$ (corresponding to $1\mu\text{m}^{3}$). The error bars denote standard deviations from mean position.

**Fig. S6. Possible DNA configuration in *C. crescentus*.**

Schematic illustration of a DNA configuration that could give rise to the observed small deviations from the linear correlation between position on the chromosome and in the cellular volume. The *ori* to *ter* strand preferably stays close to *ter*, while the *ter* to *ori* strand stays close to *ori*. The figure on the right shows the corresponding *z*-position of the genes as function of their position on the chromosome (solid curves). The dashed curve represents a perfect linear correlation. In the experimental data the deviation from the linear correlation is much smaller than shown here.

**Fig S7. Construction scheme for SAWs.**

Starting from a minimal self-avoiding walk that connects *ori* and *ter* a randomly chosen bond is deleted. If the (randomly chosen) neighboring lattice sites are free (gray) they are incorporated into the random walk. In this way, the chain is closed again and *ori* and *ter* remain at their original positions.

**Fig. S8. Flip- and crankshaft-transformations for SAWs.**

A flip of the gray bead converts the random walk 2122 (a) into 2212 (b). In (c) the gray bead is not allowed to flip. This problem is resolved by a crankshaft move that transforms $212\bar{1}2$ into $2\bar{1}212$ (d). Here, the random walk is represented by a string of symbols where, e.g. 1 represents “up”, $\bar{1}$ “down”, 2 “right”, and $\bar{2}$ “left”.

**Fig S9. 3-dimensional crankshaft-transformation.**

Three-dimensional crankshaft-transformations introduce new symbols into the random chain. In the example shown, the random walk $221\bar{2}$ is transformed into $\bar{1}211$, thus replacing a $2,\bar{2}$pair by a $1,\bar{1}$ pair. This is accomplished by the following sequence of transformations (shown from left to right): a crankshaft move ($221\bar{2}\to231\bar{3}$), followed by two bead flips ($231\bar{3}\to321\bar{3}\to32\bar{3}1$) and another crankshaft move ($32\bar{3}1\to\bar{1}211$). All moves operate on the gray beads. The black beads are fixed.

**Fig. S10. Direct comparison of the BF and the MOS method.**

Starting from a common initial configuration 1000 BF- and MOS-moves were performed to obtain 50000 different SAWs of length 2000 confined to a volume of $V=32\times32\times32$steps. The figure shows the mean *z(s)* curves obtained by these methods by averaging over the 50000 samples. The mean standard deviations are 0.07 (BF) and 0.19 (MOS) showing that the BF method produces walks that stay much closer to the initial configuration.

**Fig. S11. Comparison of the MOS algorithm with exact enumeration by using the radial density of self-avoiding walks.**

Figure A shows the density of self-avoiding loops generated by the MOS algorithm. The length of the random walks was $10\xi_{p}$for the strand connecting origin and terminus and $10\xi_{p}$for the strand connecting terminus and origin. The distance between terminus and origin is $2\xi_{p}$ (along the *z*-axis). Figure B shows the difference between the densities of the random ensemble and the exact ensemble. The MOS method reproduces the exact curves quite well except for a small region between *ori* and *ter* that is slightly underrepresented.

**Fig. S12. Comparison of the MOS algorithm with exact enumeration by using the statistics of the *z*-positions.**

The figure shows average *z-*positions of self-avoiding random walks as calculated with the MOS algorithm (red curve) and by systematic enumeration (green curve). Data are for the same parameter values as Fig. S11.
