# Supplementary figures and images for: A Geometrical Model for DNA Organization in Bacteria
Source: PLoS One. 2010 Nov 3;5(11):e13806. doi: 10.1371/journal.pone.0013806 (PMC2972204; doi:10.1371/journal.pone.0013806)

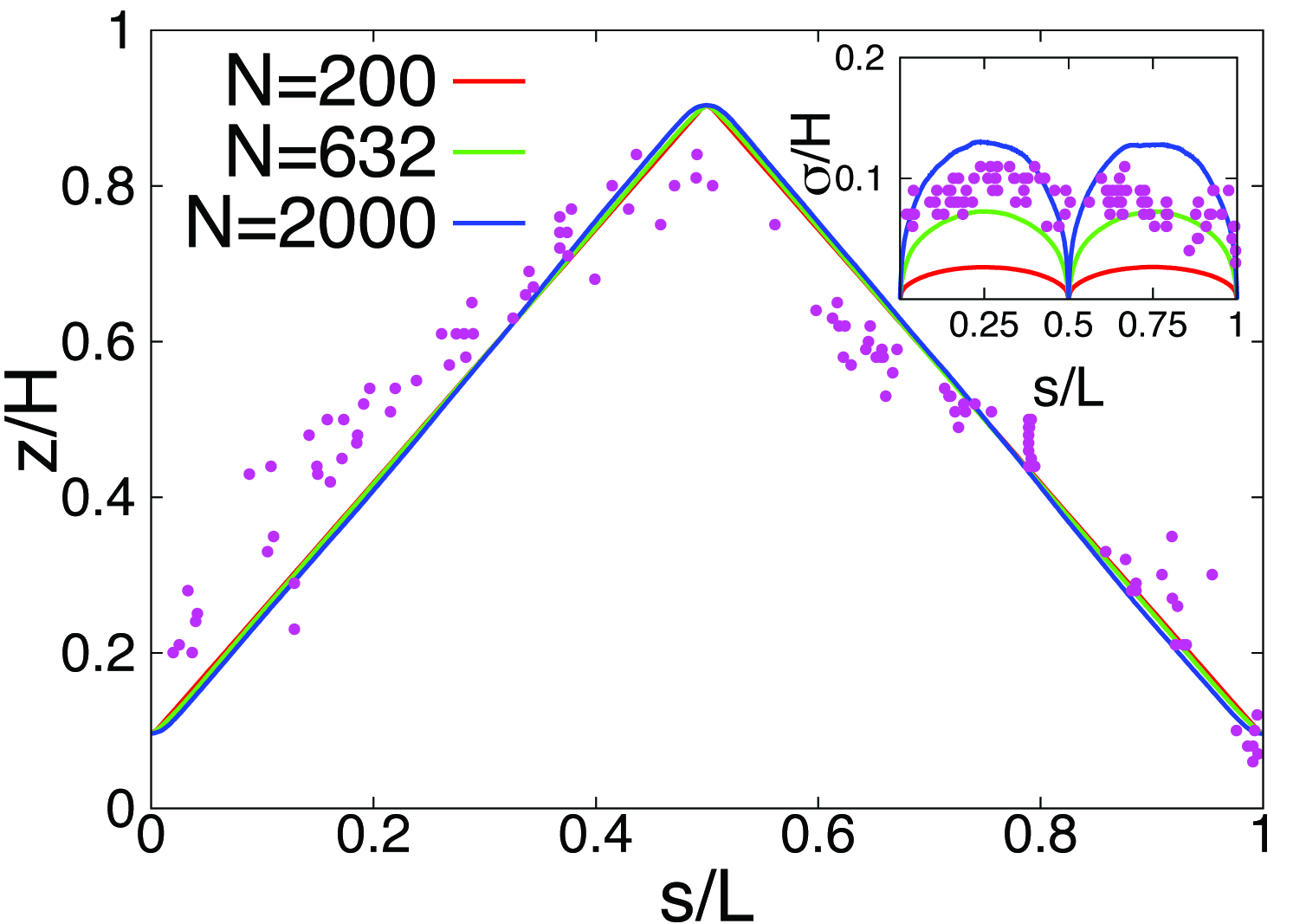

Supplement: Figure S1 — Dependence of the average DNA configuration in C. crescentus on the number of blobs as obtained from numerical simulations of compacted DNA. The z-position of an average chromosome configuration was calculated from our model in which compacted DNA is represented by a chain of blobs. The position on the chromosome is parameterized by the contour length s (measured in units of DNA length L). The configurations shown are for different number of blobs with diameter . ori and ter have fixed positions at and . (0.73 MB TIF) [file pone.0013806.s002.tif]

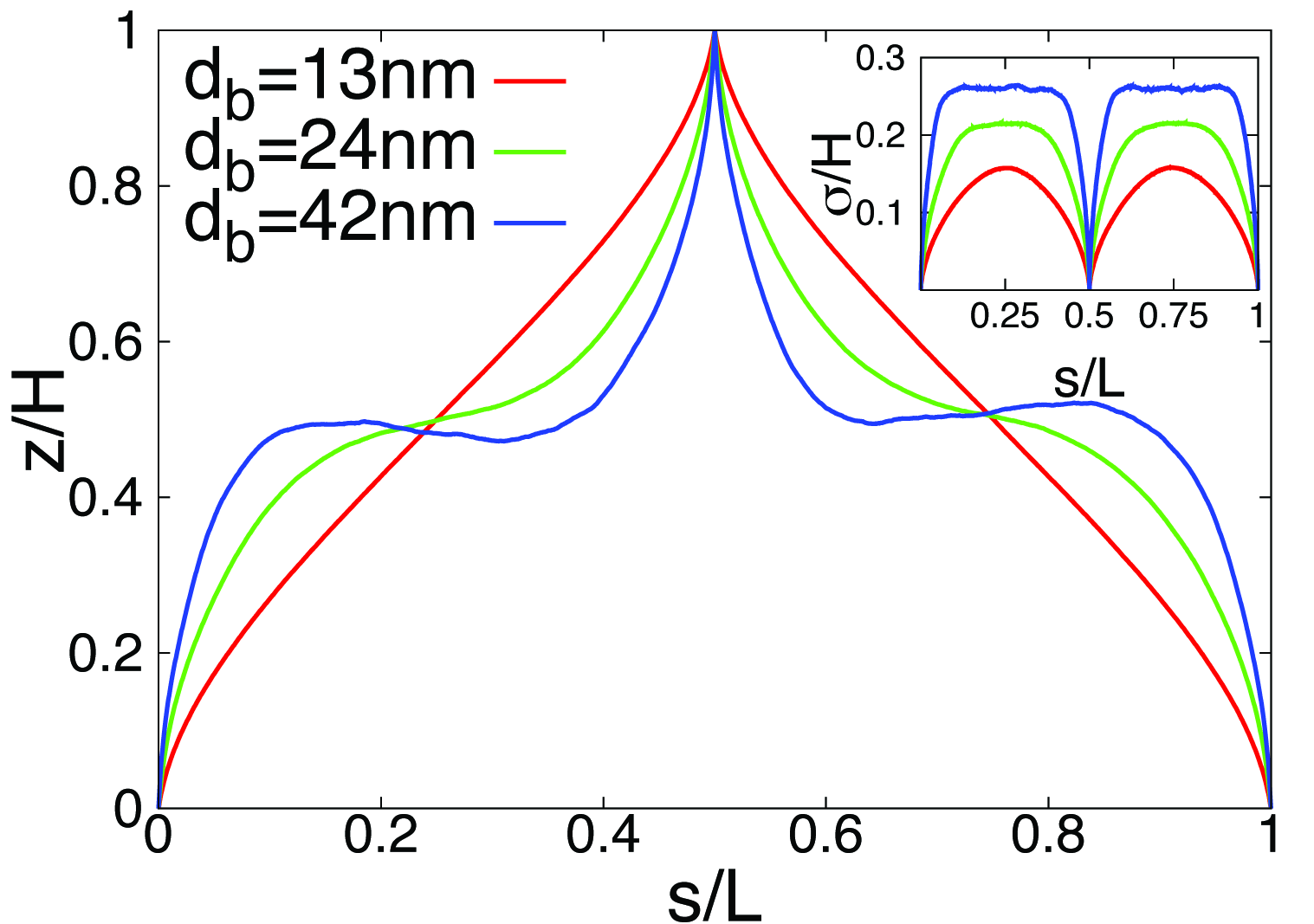

Supplement: Figure S2 — Average subcellular position of genes as function of their position on the chromosome in newborn E. coli cells as obtained from numerical simulations of compacted DNA. The figure shows the z-position of an average chromosome configuration as function of the contour length s. In our model a chain of blobs represents the compacted DNA. Configurations shown are for different blob diameters by assuming a constant number (2000) of blobs. ori and ter are positioned at opposite cell poles ( and ). The insets show the (rescaled) standard deviations from the mean configurations as function of s. (0.74 MB TIF) [file pone.0013806.s003.tif]

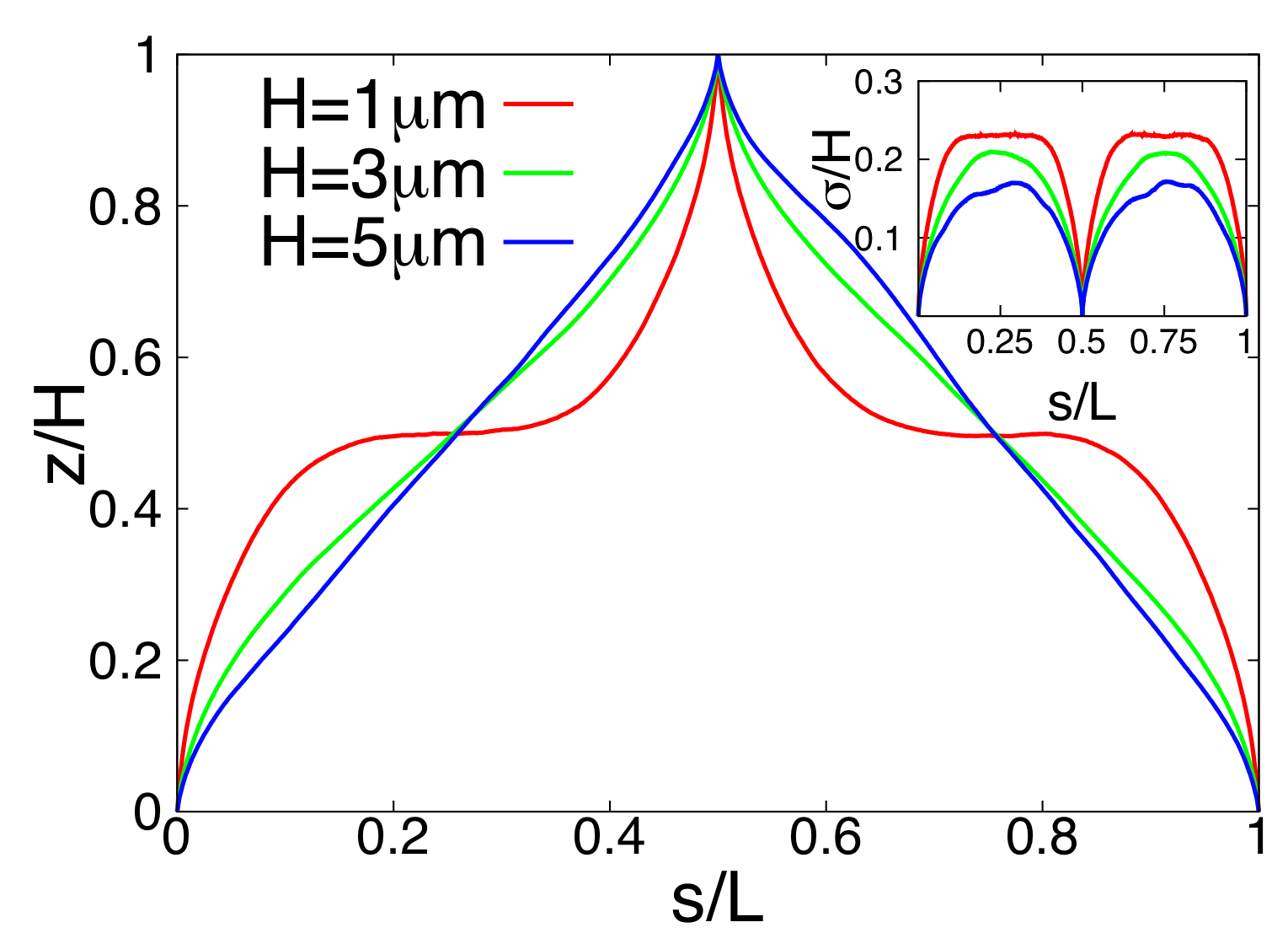

Supplement: Figure S3 — Dependence of the average chromosome configuration in newborn E. coli cells on the cellular volume. The z-position (as function of the contour length s) of an average chromosome configuration was calculated from our model in which compacted DNA is represented by a chain of blobs. In the figure the volume is varied by changing the length of the cells () by keeping the aspect ratio of the cross section fixed. Chromosome length is varied together with the volume such that the DNA density in the volume remains constant. Furthermore, the number of compaction proteins is assumed to be growth-rate independent. The chromosome is represented by 2000 blobs with a volume-dependent diameter (). Ori and ter are positioned at opposite cell poles ( and ). The insets show the (rescaled) standard deviations from the mean configurations as function of s. (0.22 MB TIF) [file pone.0013806.s004.tif]

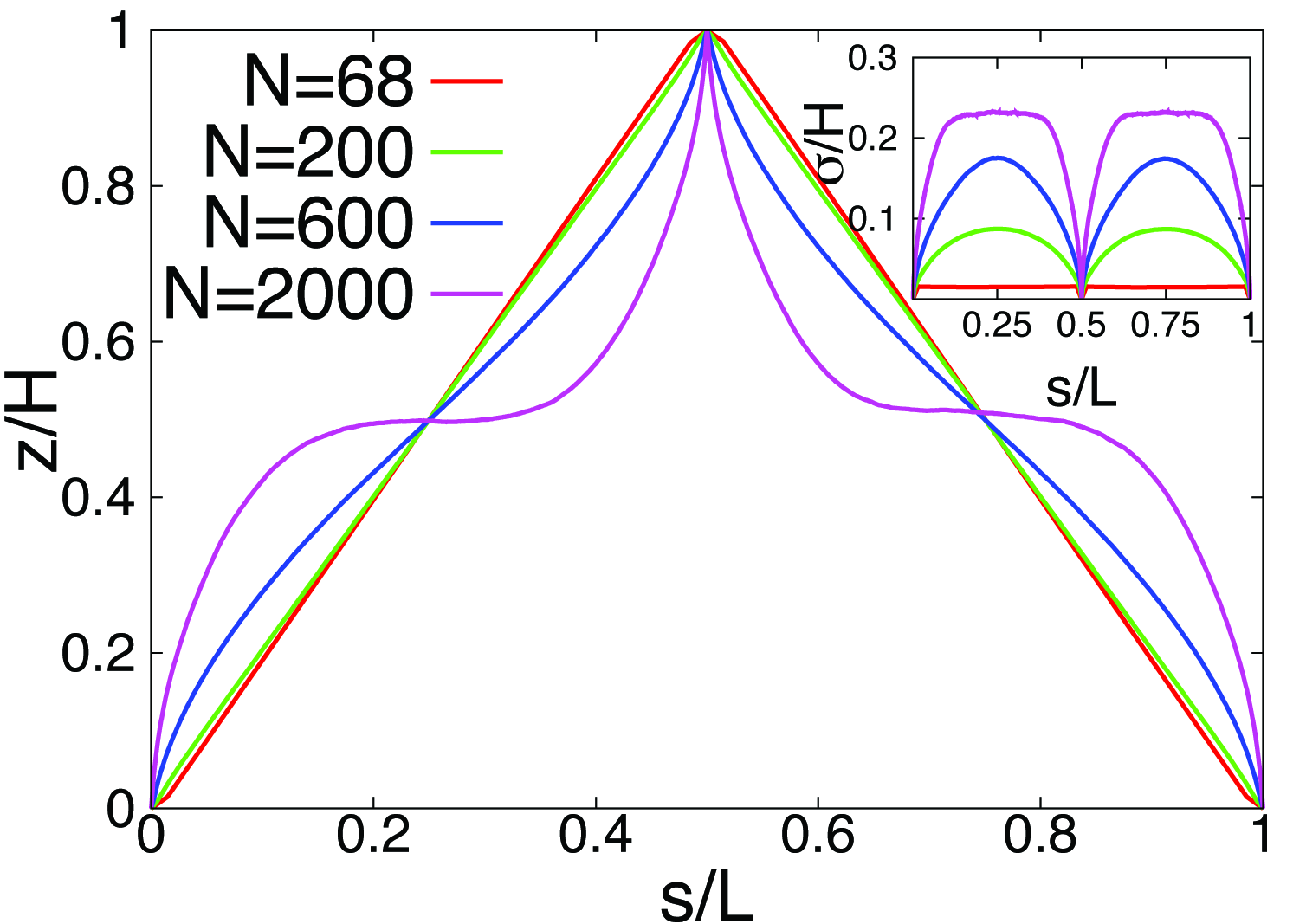

Supplement: Figure S4 — Dependence of the average DNA configuration on the number of blobs for newborn E. coli cells as obtained from numerical simulations of compacted DNA. The figure shows the z-position of an average chromosome configuration as function of the contour length s. The configurations shown are for different number of blobs with diameter . Ori and ter are positioned at opposite cell poles ( and ). (0.76 MB TIF) [file pone.0013806.s005.tif]

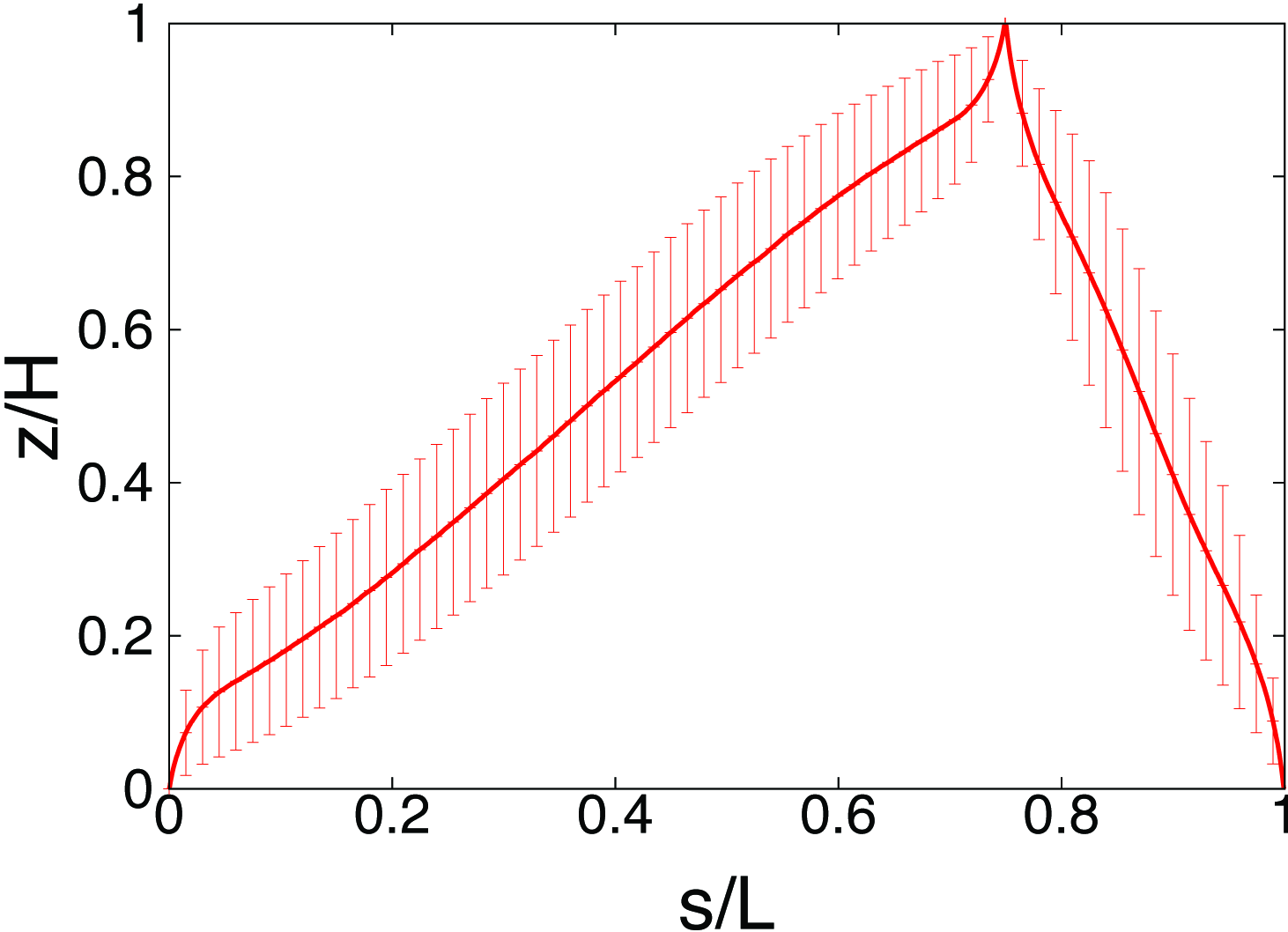

Supplement: Figure S5 — Average DNA configuration in a newborn E. coli mutant cell where ori is located at 6 o'clock and ter at 3 o'clock on the chromosome. Both ori and ter are located at opposite cell poles ( and ). In the simulations the chromosome is represented by a self-avoiding chain of blobs with diameter . The chain consists of 2000 blobs: 1500 blobs for the strand connecting ori and ter and 500 blobs for the strand connecting ter and ori. Cell size is (corresponding to ). The error bars denote standard deviations from mean position. (0.69 MB TIF) [file pone.0013806.s006.tif]

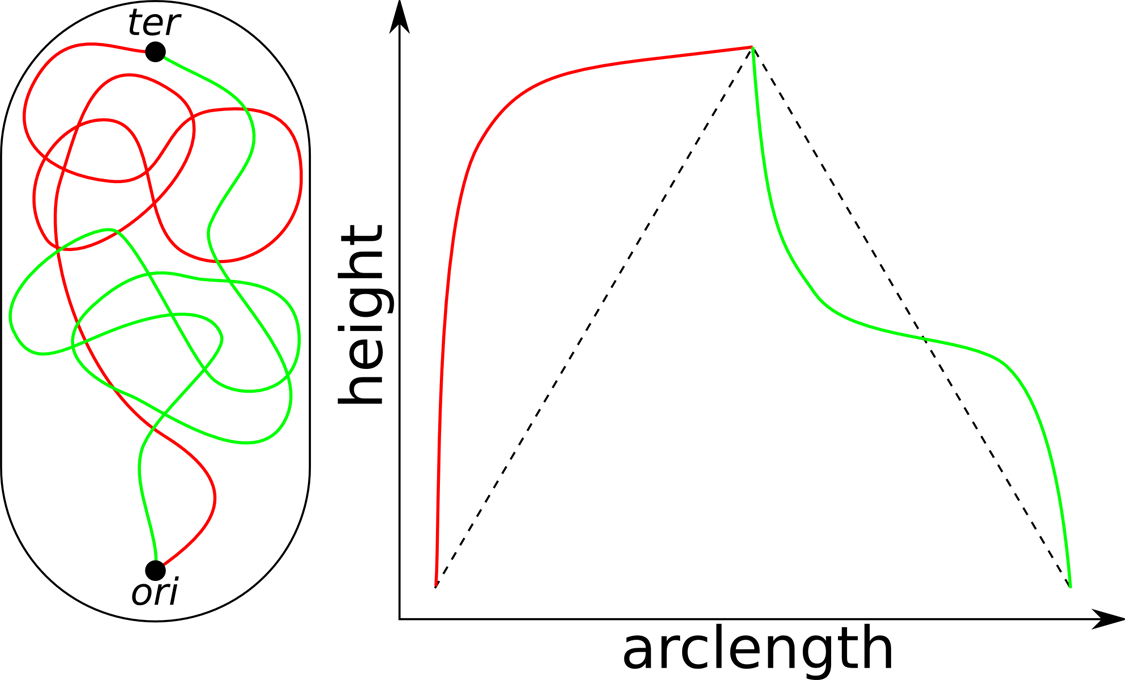

Supplement: Figure S6 — Possible DNA configuration in C. crescentus. Schematic illustration of a DNA configuration that could give rise to the observed small deviations from the linear correlation between position on the chromosome and in the cellular volume. The ori to ter strand preferably stays close to ter, while the ter to ori strand stays close to ori. The figure on the right shows the corresponding z-position of the genes as function of their position on the chromosome (solid curves). The dashed curve represents a perfect linear correlation. In the experimental data the deviation from the linear correlation is much smaller than shown here. (0.14 MB TIF) [file pone.0013806.s007.tif]

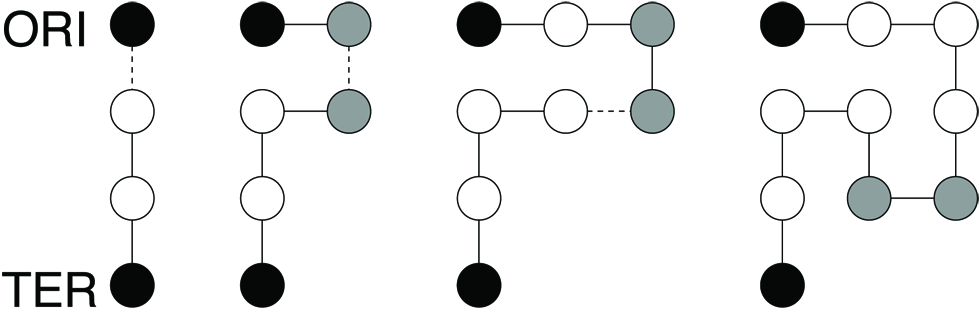

Supplement: Figure S7 — Construction scheme for SAWs. Starting from a minimal self-avoiding walk that connects ori and ter a randomly chosen bond is deleted. If the (randomly chosen) neighboring lattice sites are free (gray) they are incorporated into the random walk. In this way, the chain is closed again and ori and ter remain at their original positions. (0.65 MB TIF) [file pone.0013806.s008.tif]

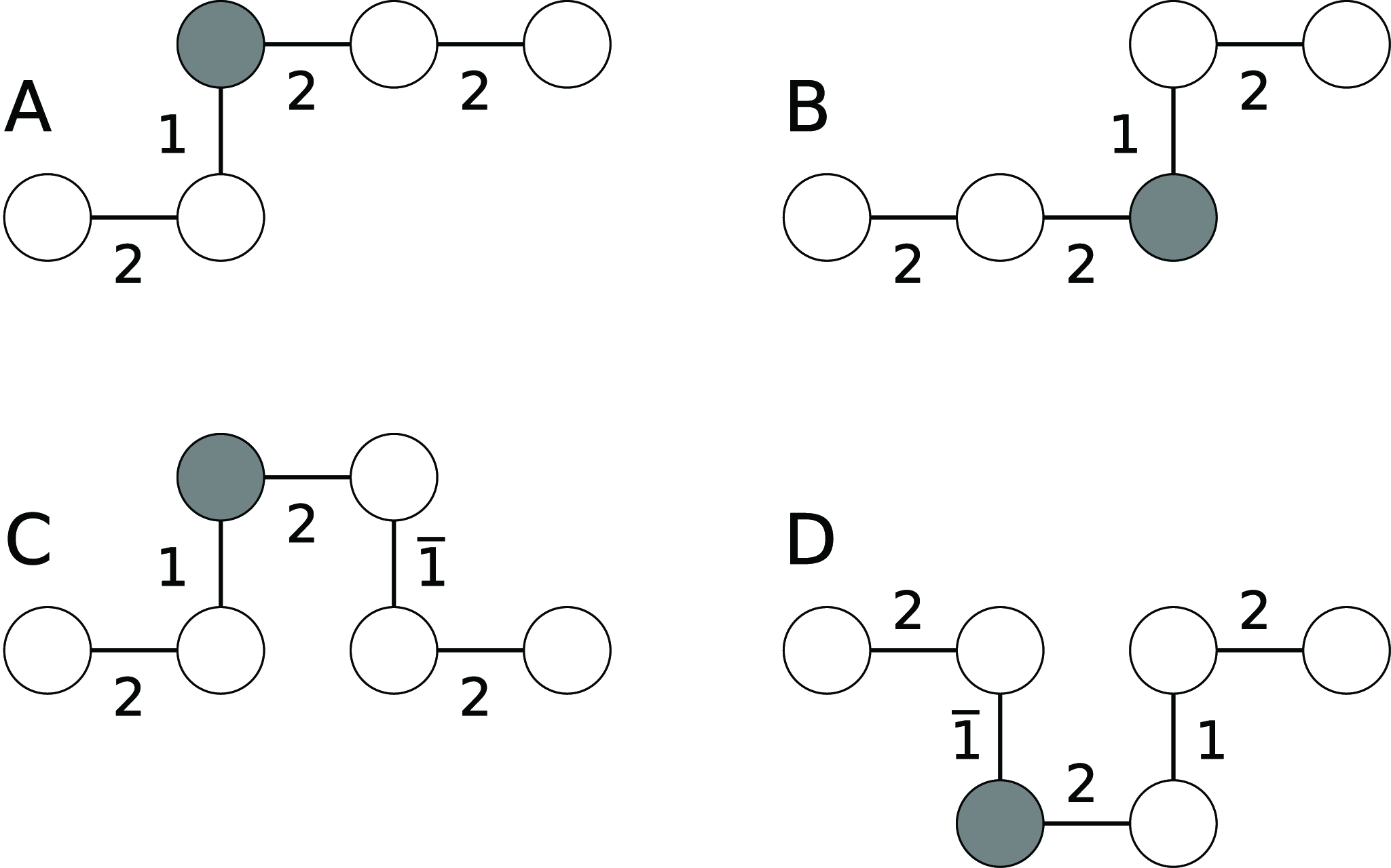

Supplement: Figure S8 — Flip- and crankshaft-transformations for SAWs. A flip of the gray bead converts the random walk 2122 (a) into 2212 (b). In (c) the gray bead is not allowed to flip. This problem is resolved by a crankshaft move that transforms into (d). Here, the random walk is represented by a string of symbols where, e.g. 1 represents “up”, “down”, 2 “right”, and “left”. (0.70 MB TIF) [file pone.0013806.s009.tif]

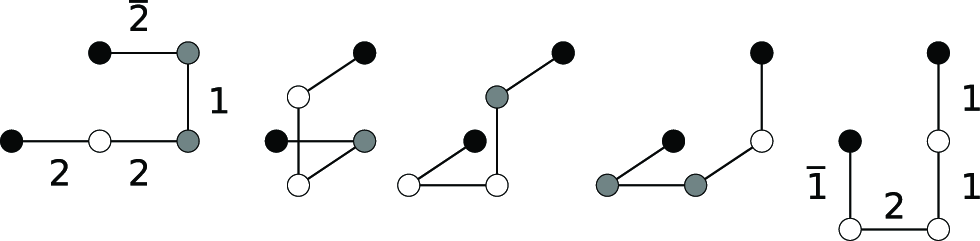

Supplement: Figure S9 — 3-dimensional crankshaft-transformation. Three-dimensional crankshaft-transformations introduce new symbols into the random chain. In the example shown, the random walk is transformed into , thus replacing a pair by a pair. This is accomplished by the following sequence of transformations (shown from left to right): a crankshaft move (), followed by two bead flips () and another crankshaft move (). All moves operate on the gray beads. The black beads are fixed. (0.60 MB TIF) [file pone.0013806.s010.tif]

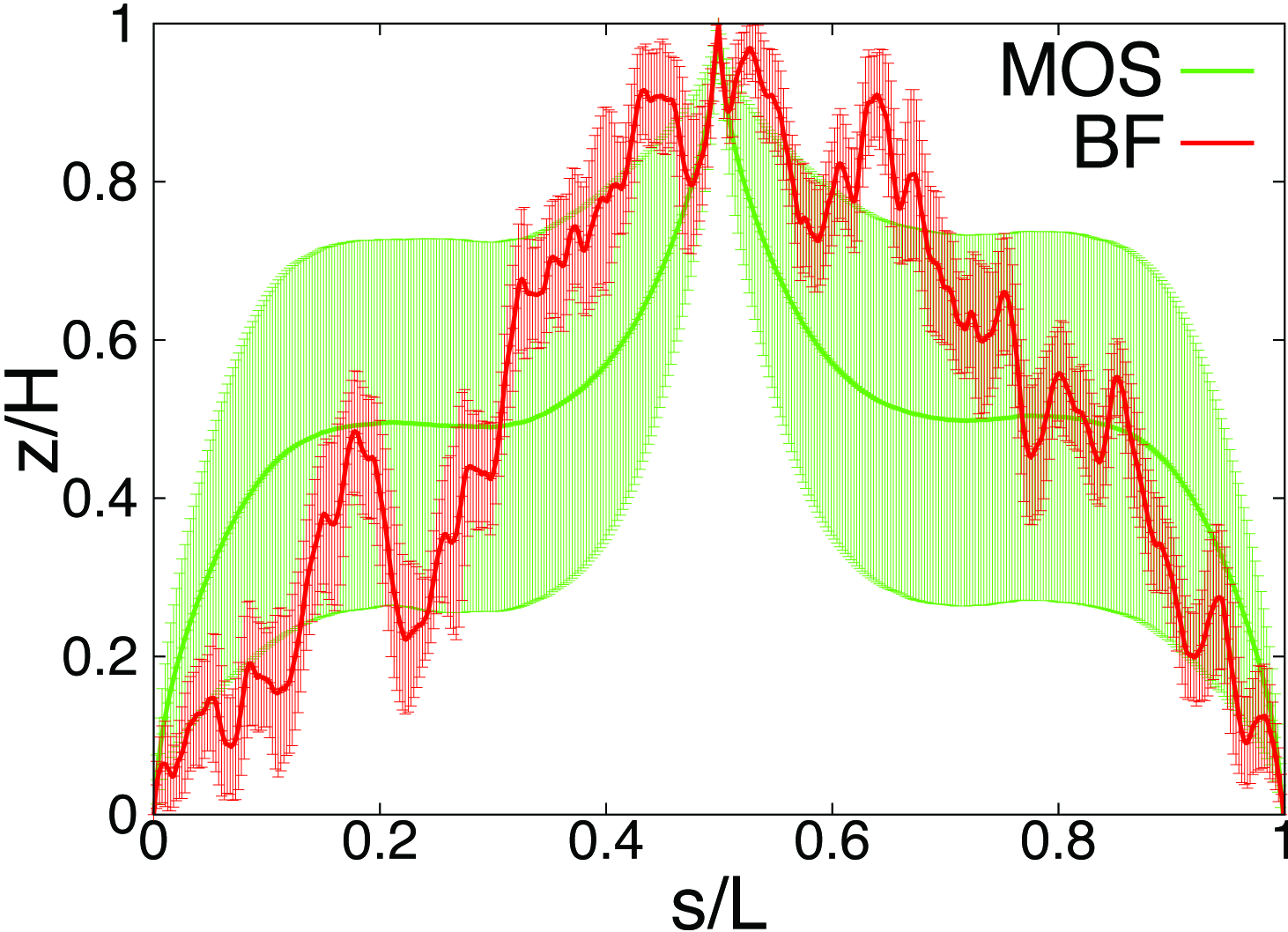

Supplement: Figure S10 — Direct comparison of the BF and the MOS method. Starting from a common initial configuration 1000 BF- and MOS-moves were performed to obtain 50000 different SAWs of length 2000 confined to a volume of steps. The figure shows the mean z(s) curves obtained by these methods by averaging over the 50000 samples. The mean standard deviations are 0.07 (BF) and 0.19 (MOS) showing that the BF method produces walks that stay much closer to the initial configuration. (1.04 MB TIF) [file pone.0013806.s011.tif]

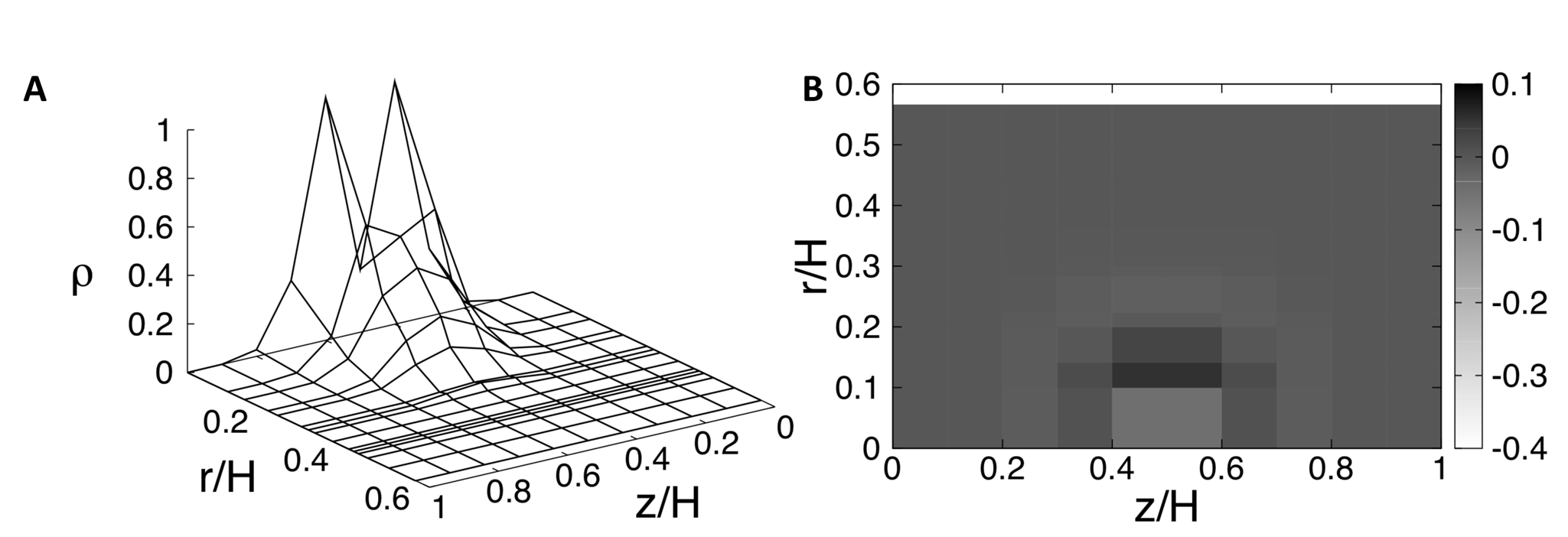

Supplement: Figure S11 — Comparison of the MOS algorithm with exact enumeration by using the radial density of self-avoiding walks. Figure A shows the density of self-avoiding loops generated by the MOS algorithm. The length of the random walks was for the strand connecting origin and terminus and for the strand connecting terminus and origin. The distance between terminus and origin is (along the z-axis). Figure B shows the difference between the densities of the random ensemble and the exact ensemble. The MOS method reproduces the exact curves quite well except for a small region between ori and ter that is slightly underrepresented. (0.24 MB TIF) [file pone.0013806.s012.tif]

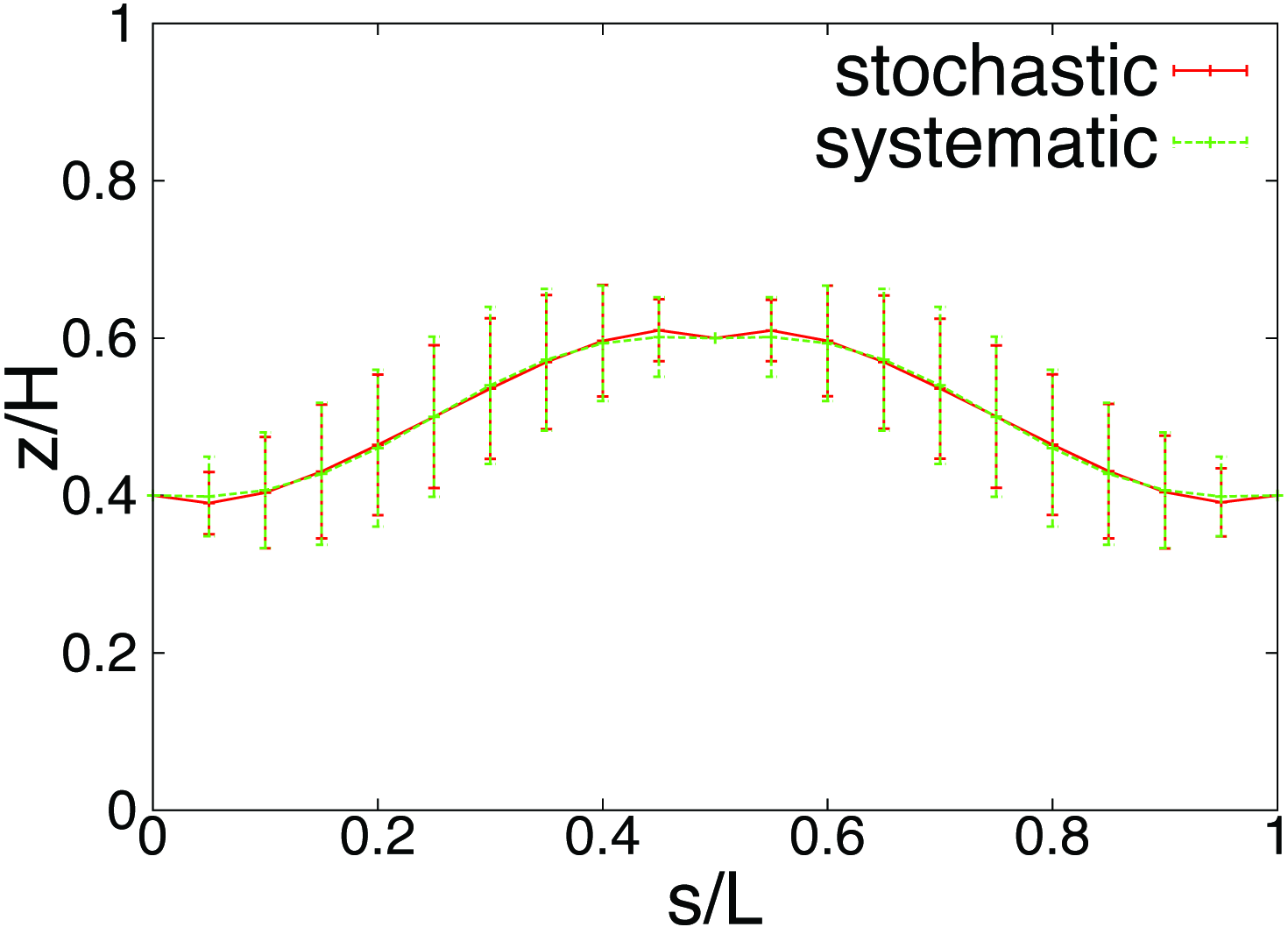

Supplement: Figure S12 — Comparison of the MOS algorithm with exact enumeration by using the statistics of the z-positions. The figure shows average z-positions of self-avoiding random walks as calculated with the MOS algorithm (red curve) and by systematic enumeration (green curve). Data are for the same parameter values as Fig. S11. (0.68 MB TIF) [file pone.0013806.s013.tif]
